# Tailored online eating disorder prevention and health promotion for women: results of a dissemination trial

**DOI:** 10.3389/fpsyt.2025.1731066

**Published:** 2026-01-22

**Authors:** Barbara Nacke, Dennis Görlich, Ina Beintner, Bianka Vollert, Juliane Schmidt-Hantke, C. Barr Taylor, Corinna Jacobi

**Affiliations:** 1 Institute of Clinical Psychology and Psychotherapy, Faculty of Psychology, Technische Universität Dresden, Dresden, Germany; 2 Institute of Biostatistics and Clinical Research, Universität Münster, Münster, Germany; 3 Department of Psychiatry, Stanford University School of Medicine, Stanford, CA, United States

**Keywords:** eating disorders, health promotion, internet-based intervention, population-based prevention, prevention, public health, women’s health

## Abstract

**Introduction:**

Internet-based interventions are effective in the prevention of eating disorders (EDs) but are rarely translated from controlled study settings into practice. This study aimed to assess short- and long-term outcomes and adherence of a suite of internet-based screening and ED prevention programs.

**Methods:**

In a 5-arm non-randomized dissemination trial, internet-based ED prevention interventions were offered to women recruited from the general population (*N* = 3,654). Each arm offered a different version of the ED prevention program, tailored for populations at different levels of risk. The interventions comprised 4 to 12 weekly modules based on cognitive-behavioral principles, including psychoeducation, exercises to promote body image and balanced eating, and—if applicable—to reduce ED symptoms. Primary outcome was the change in weight concerns from pre to post, using t-tests and completer data. Secondary outcomes included ED symptoms, eating habits, self-esteem and quality of life.

**Results:**

Pre-post within-subject comparisons in the completer sample showed significant reductions in weight concerns in 4 of 5 study arms (effect sizes between *d* = -0.45 and *d* = -0.94). ED symptoms were reduced and the ability to eat intuitively was improved in all study arms, with some effects persisting up to 12 months. Assessment drop-out ranged from 60.6% to 78.1% at post, and between 18.0% and 44.0% of participants completed the whole intervention.

**Discussion:**

The trial demonstrates the feasibility of reaching different risk groups for prevention with a combined screening and tailored interventions as well as feasibility of larger scale dissemination of the interventions in the general population.

**Clinical trial registration:**

http://www.isrctn.org, identifier ISRCTN13716228.

## 1 Introduction

Eating disorders (EDs) are serious mental disorders associated with high morbidity and mortality (1), affecting 8.4% of adult females and 2.2% of adult males worldwide (2). EDs are associated with marked psychosocial impairment, psychiatric comorbidity, and poor quality of life (3, 4). They are characterized by binge eating behaviors, and/or methods to control weight such as purging, excessive exercise, diet pill use, and food restriction. Given their high prevalence and significant impact, scalable and broadly applicable preventive interventions are of great public health importance.

Research in the past few decades has identified a number of modifiable risk factors including body dissatisfaction, dieting, negative affect, weight and shape concerns and thin-ideal internalization (5). Furthermore, there is an overlap between risk factors for eating disorders and overweight and obesity (6, 7) and eating disturbances occur more frequently in women with overweight and obesity than in women with normal weight or underweight (8). Obesity is a risk factor for binge eating disorder (BED) and possibly bulimia nervosa (BN) (5) and, in turn, disordered eating behaviors such as dieting, purging, laxative/diuretic use, and binge eating contribute to excess weight gain (9, 10). As EDs and overweight share risk factors, addressing these and their interactions in preventive interventions could facilitate healthy weight management without increasing weight or shape concerns or facilitating disordered eating (11–13).

Fortunately, many studies have shown that preventive internet-based interventions can reduce ED risk factors and symptoms (14–18) and even reduce ED onset (19, 20). However, while most of this research of EDs has focused on adolescents and young women (21, 22), disordered eating, dieting, and body dissatisfaction are also prevalent in middle-aged and older women (8, 23).

Over the past decade, our research group has developed a suite of internet-based interventions targeting different ED risk groups for selective prevention (24–28) and general populations for universal prevention (29). These interventions have been delivered digitally as self-help and/or as guided self-help to facilitate ease of access, anonymity and provide service at low cost. The interventions proved to be well accepted and efficacious in younger women, e.g., college-age students (25–27), but two interventions also showed promise for improvement of body image and disordered eating in all age groups (everyBody Basic and everyBody Fit) (24, 29).

To date, only few studies evaluated the effectiveness, reach and dissemination of internet-based ED prevention programs in real world settings (e.g., education systems, healthcare systems, social and traditional media). These programs have mostly been implemented in schools and colleges, and demonstrated successful implementation (30–32). However, age groups past the college age affected by body dissatisfaction and other frequent ED risk factors were not included in these studies.

Increasing reach of prevention programs is crucial for the prevention of ED and might be more important than solely increasing the efficacy of programs in terms of their public health impact (33). Accordingly, while universal prevention programs often produce smaller effects than selective prevention (16), they might be better suited for broader dissemination and as population-based approaches (34).

The aim of the current dissemination study therefore was to provide and evaluate a population-based approach for ED prevention targeting shared risk factors of EDs and overweight. The intervention was tailored to individual ED risk status and ED symptoms and combines ED prevention and health promotion for balanced eating and exercise in women of different age groups. Specifically, we aimed to evaluate short- and long-term effectiveness and to examine demographic characteristics of participants, feasibility, and adherence.

## 2 Methods

### 2.1 Participants

We included women aged 18 and older, interested in participating in an online program to improve their body image, with access to the internet and who gave informed consent online. We excluded women who reported currently receiving psychotherapy for EDs or who had received ED treatment in the past six months. We also excluded women who met the diagnostic criteria for full-syndrome AN, BN or BED and referred them to treatment, as well as women with a body mass index (BMI) below 18.5 kg/m². Participants had to provide an e-mail address to be enrolled in the trial, but did not need to provide their full name.

Participants were recruited via various means directed at a general audience (e.g., press releases, online and print newspaper articles, TV reports, promotional postcards in malls), through health insurances which offered the program as part of their internet-based prevention portfolio, through medical practices (e.g., promotional postcards and posters in waiting rooms) and through means directed at students (e.g., announcement in lectures, posters around campus). Publications about the study included a wide variety of online (including social media), face-to-face and print media to reach the target group of women of all ages from German speaking countries. The intervention was announced as a free online intervention for women who wanted to improve body image and reduce weight and shape concerns. Recruitment took place between November 2016 and May 2019. Students were offered course credits for participation but other participants were initially not compensated for study participation. Within the first months of recruitment, internal data quality reports revealed high drop-out rates for post-intervention and follow-up assessments. Therefore, to improve adherence to the study protocol, participants who completed the post-intervention assessment were entered into a raffle. Each month, four 25€ gift cards were provided.

### 2.2 Study design and procedure

**Figure 1:**
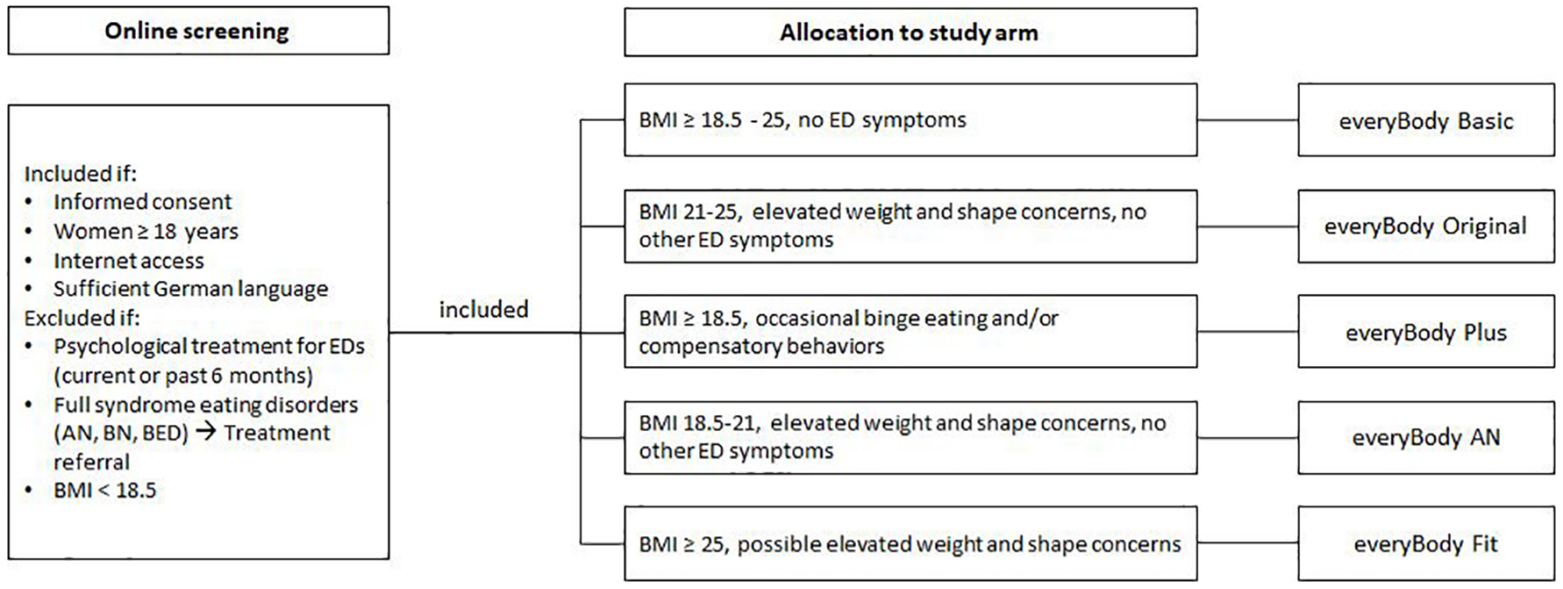
Participants were screened and included participants were allocated to study arm based on BMI and ED symptoms.

The design of the study was a nonrandomized, parallel-group interventional design. Before enrollment in the intervention, participants were screened for EDs and excluded if exclusion criteria were fulfilled (see **Figure 1**). Based on the screening information, participants were allocated to one of the five study arms (see **Figure 1**). The allocation of participants was based on their BMI, the presence of subthreshold (<1/week) binge eating/purging, and the presence of elevated weight and shape concerns defined as a score larger than 42 on the weight and shape concerns scale (WCS) (35).

Time between screening and baseline assessment varied between 0–2 weeks. Following inclusion, participants completed baseline assessments (T1) and received access to the intervention they had been assigned to. Further assessments took place at mid-intervention (Tmid), post-intervention (T2), 6-month follow-up (T3), and at 12-month follow-up (T4). Mid-intervention assessment was administered 4 weeks after baseline (except Basic). Post-intervention assessments were administered 4, 8, 10 or 12 weeks after baseline, depending on duration of the intervention in each study arm (see **Table 1**).

**Table 1:** Characteristics of the five study arms.

Feature	everyBody study arm				
	Basic	Original	Plus	AN	Fit
ED symptoms	None	Elevated weight and shape concerns, no other ED symptoms	Elevated weight and shape concerns, occasional binge eating and/or compensatory behaviors	Elevated weight and shape concerns, no other ED symptoms	Possible elevated weight and shape concerns, no other ED symptoms
BMI	18.5-25	21-25	≥ 18.5	18.5-21	> 25
Duration (weeks)	4	8	8	10	12
Aims	Promoting balanced eating and exercise habits, body image	Improving body image, promoting balanced eating and exercise habits	Improving body image, establishing balanced eating and exercise habits, improving self-esteem, reducing compensatory behaviors	Improving body image, establishing balanced eating and exercise habits, improving self-esteem, reducing dietary restraint	Healthy weight regulation, promoting balanced eating and exercise habits, improving self-esteem and body satisfaction
Moderated discussion groups	No	Yes	Yes	Yes	Yes
Individualized weekly feedback	No	No	Yes	Yes	No
Accompanying diaries	No	Yes	Yes	Yes	Yes

All assessments as well as the interventions were conducted online on an intervention platform provided by Minddistrict (Minddistrict, Amsterdam, The Netherlands), a company specialized in providing online platforms for psychological interventions and research. To be enrolled in the trial and to get access to the assessments and intervention, each participant created a password-protected account on the platform.

This trial has been registered at ISRCTN (ISRCTN13716228) and has been approved by the ethics board of TU Dresden (EK 83032016). The study protocol describing the methods and planned analyses has been published (36).

### 2.3 Intervention

#### 2.3.1 Content and dosage

For the purpose of this dissemination study, the intervention was adapted from StudentBodies interventions (25–27, 29, 37) and StayingFit (24), all evaluated in previous trials. The adapted version comprises a combined online screening and suite of five tailored, evidence-based prevention programs (“everyBody”), accessible via web browser. Each study arm targets different risk factors presumed to lead to or maintain eating pathology and different stages of risk for eating disorders and/or overweight and obesity (see **Figure 1**). The characteristics of the five programs are summarized in **Table 1**.

The intervention content of all these programs is based on cognitive-behavioral principles and aims to improve cognitive, affective and behavioral outcomes. These include psychoeducation, self-monitoring, a personal journal and behavioral exercises. The content focuses on balanced eating and exercise, intuitive eating, self-esteem, dealing with “forbidden foods” and binge eating/purging, improving body image, coping with stress and negative emotions. Sections on intuitive eating promoted eating based on satiety and hunger, reduction of emotional eating and self-permission to eat whatever foods are desired when hungry. The contents of the everyBody interventions are described in more detail in previous publications (24, 26, 38).

Participants were encouraged to complete one session per week, to allow time for consolidation and practice. Each session took 20 to 60 minutes to complete. We added a one-week break between releasing sessions, making it impossible to complete the program ahead of the intended schedule, but there was no upper time limit for completing the intervention. All participants received weekly reminders during the intended duration of the intervention if they did not complete a session. They also received generic weekly e-mail notifications for uncompleted assessments, diary entries, unread messages on the platform and uncompleted sessions.

#### 2.3.2 Discussion groups and diaries

Four study arms (Original, Plus, AN, and Fit) were supplemented by moderated discussion groups to promote the exchange of experiences and thoughts with other participants. Discussion groups were implemented as multi-participant, asynchronous group chats, in which participants from the same study arm were able to communicate with each other. When completing a session, participants were automatically invited to discussion groups themed around the session topics. In each discussion group, the moderator, i.e., a trained psychologist, posted an opening question and moderated the following discussions. The same study arms were supplemented with diaries to record eating habits, exercise behavior, as well as thoughts and feelings related to body image and disordered eating, which were recommended for daily or weekly use.

#### 2.3.3 Individualized feedback

In two arms (Plus and AN), participants received weekly individualized feedback by a coach. Coaches were psychologists (bachelor’s or master’s degree), who received a 1.5h training and continuous supervision by a licensed clinical psychologist specialized in EDs (IB). Based on their entries in diaries and sessions as well as comments in the discussion group, participants received feedback encouraging their motivation to change, helping with cognitive restructuring, suggestions for behavior change, and additional psychoeducation, based on the CBT approach for EDs (39). Participants were encouraged to answer directly to their coach, but messages from the coach were usually limited to one per week. Coaches also provided technical support, if needed.

### 2.4 Measures

#### 2.4.1 Screening

A prediction algorithm was applied to exclude potential AN, BN and BED cases from the trial. The online screening included 13 items addressing BMI, weight and shape concerns, restrained eating, loss of control eating and overeating and was previously validated in an independent sample of 215 German women. The screening algorithm had shown excellent classification properties, detecting 98.4% and 91.9% of AN and BN cases (true positives), respectively, as well as good properties in detecting 87.1% BED cases (40). In the current study, the screening algorithm initially categorized a BMI of 41.4 or higher as indicative of BED, regardless of any other symptoms, meaning women with a BMI of 41.4 or higher would be all excluded by the algorithm. To avoid false exclusions, we evaluated all participants with BMI > 41.4 individually for clinically relevant overeating and loss of control eating.

#### 2.4.2 Socio-demographic information

Socio-demographic characteristics of participants, such as year of birth, occupation, relationship status, and self-reported height and weight were assessed. Presence of lifetime diagnoses were assessed via self-report of participants.

#### 2.4.3 Primary and secondary outcomes

The primary outcome was weight and shape concerns. Secondary outcomes were ED pathology, eating styles and habits, self-esteem, and quality of life.

##### 2.4.3.1 Weight and shape concerns

Weight and shape concerns were assessed using the Weight Concerns Scale (WCS) (35, 41). The WCS has excellent test–retest reliability of r = .75 for a 12-month interval (English version) (42) and r = .95 for a 7-day interval (German version) (35). The German version showed excellent convergent and discriminant validity (35).

##### 2.4.3.2 ED pathology

ED pathology was assessed using the Eating Disorder Examination-Questionnaire global score [EDE-Q; 43, 44], a 22-item questionnaire covering restrained eating behaviors, eating concerns, and weight and shape concerns. It has good convergent and discriminant validity, shown in a non-clinical sample, as well as good to excellent internal consistency (45).

Core ED symptoms (i.e., number of binge eating episodes in the past four weeks, number of compensatory and weight control behaviors in the past four weeks) were measured using 5 items covering core diagnostic ED behaviors of the EDE-Q (44), omitting the number of days with objective binge eating. Three items assessing the presence of fasting, use of appetite suppressants, and use of diuretics were added (e.g., “Over the past 28 days, how many times have you taken diuretics as a means of controlling your shape or weight?”. Filter questions were added where participants indicated whether the respective symptom had been present during the past 28 days, before they reported the number of incidents. These answers were used to report the presence vs. absence of core symptoms.

##### 2.4.3.3 Eating styles and habits

Intuitive eating was assessed using the Intuitive Eating Scale (IES) (46, 47). The scale assesses three aspects of intuitive eating: ‘‘unconditional permission to eat (when hungry and what food is desired)” (UPE subscale), “eating for physical rather than emotional reasons’’ (EPR subscale), ‘‘reliance on internal hunger and satiety cues to determine when and how much to eat’’ (RIH subscale). Higher total scores indicate higher levels of intuitive eating. Factorial validity, internal consistency, test-retest reliability, as well as convergent and discriminant validity were shown in a sample of female university students (46).

Fruit and vegetable intake was assessed by four items. Participants reported how many portions of fresh fruit, fresh vegetables, frozen vegetables, and smoothies had been consumed in the past 7 days on a 7-point response scale ranging from 0 to 6 (0, none during the past 7 days; 1, One to three portions during the last 7 days; 2, four to six portions during the last 7 days; 3, about one portion per day; 4 – about two portions per day; 5, about three portions per day; 6, four or more portions per day), e.g., “How many fist-sized portions of fruit did you eat in the last 7 days?” A portion of smoothie or juice was defined as 200 ml (6.8 US fl. oz.) in the instructions. These items had been used in previous studies on the intervention [e.g., 24], but were not assessed for validity or reliability.

##### 2.4.3.4 Other measures of (mental) health

Self-esteem was assessed using the Rosenberg Self-Esteem Scale, which measures positive and negative aspects of self-evaluation (RSE) (48, 49). Internal consistency, test-retest reliability and convergent and discriminant validity have been shown in several samples (49–51).

Quality of Life was assessed with the Assessment of Quality of Life-8D (AQoL-8D) (52), using the overall utility score of the scale. Factorial, convergent, predictive, and content validity, as well as internal consistency and test-retest reliability have been shown in Australian and US samples so far (53, 54).

Depressive symptoms were assessed with the 9-item depression module of the Patient Health Questionnaire (PHQ-9) (55, 56) Validity and reliability of the scale have been shown in samples of the German general population (57, 58). The 7-item generalized anxiety module of the Patient Health Questionnaire (GAD-7) (59) was used to assess symptoms of general anxiety disorder. Its validity and reliability has been shown in a sample of the German general population (60). Depressive symptoms and symptoms of generalized anxiety were assessed as moderators, but are only used as predictors for multiple imputation purposes within this publication.

An overview of all measures, including moderators and mediators to outcome and adherence, and their time points of assessment has been published in the study protocol (36).

#### 2.4.4 Adherence

Adherence to the intervention (completed sessions) and engagement with the intervention (diary entries, use of discussion group, number of messages to moderator) was extracted from study data and platform logfiles.

### 2.5 Adverse events

The following adverse events (AE) were defined for this trial: AE1) onset of binge eating and/or compensatory behaviors between screening and follow-up-period in participants who did not show these symptoms at screening; AE2) diagnostic threshold of frequency of binge eating and/or compensatory behaviors is met during the follow-up-period by participants with prior subthreshold symptoms; AE3) BMI drops below 18.0 kg/m^2^ during the follow-up-period; and AE4) participant reports inpatient treatment for ED during the follow-up-period.

### 2.6 Data management and statistical analyses

#### 2.6.1 Data collection

Primary data was collected on the Minddistrict platform. Participants provided self-report assessment within the Minddistrict platform. Data was downloaded for data analysis as CSV files. Data preparation, data cleaning, as well as calculation of derived variables and scores, and data analysis was performed in the SAS Software (Version 9.4, SAS Inc., Cary, NC, USA). Data was monitored for completeness during the trial by DG (University of Münster).

General principles of data analysis in the project are described by Görlich and Faldum (61). Furthermore, data analysis followed accepted guidelines such as the ICH E9 “Statistical Guidelines for Clinical Trials” (62).

#### 2.6.2 Analysis sets

The full analysis set includes all participants assigned to a study arm who completed at least the baseline assessment. Intention-to-treat analyses (ITT) were performed on the full analysis set based on the assigned study arm. The completer set includes participants who completed the questionnaires for the respective analyses in the mid assessments, post assessments, 6-months and/or 12-months follow-ups. The per-protocol set is defined by all participants included in the trial who did not exhibit major protocol violations. Protocol violations were defined by any violation of inclusion criteria with respect to the assigned study arm, i.e. participants received the wrong intervention or should have been excluded from the beginning.

#### 2.6.3 Sample size calculation

An a-priori power analysis and sample size determination was performed (36). We planned to detect an intervention effect of *d* = 0.2 in a two-sided one-sample pre-post test. A total sample size of 2080 women in all study arms would have had to be enrolled in the study and complete post-intervention assessments to reach a power of 80% in the study arm with the lowest expected recruitment, given the prevalence rates of the varying symptoms in each study arm described in **Table 1**. A-priori, a drop-out rate of 50% was assumed, based on the most recent everyBody studies with drop-out rates of 54% (24) and 45% (29), respectively and a total of 4,160 women was set as recruitment aim.

#### 2.6.4 Data analysis strategy

The statistical analysis plan was finalized before the data analysis was performed.

Each study arm was analyzed separately with two-sided statistical tests with a separate significance level of α = 0.05 for each primary confirmatory null hypothesis. Direct comparisons between study arms were neither planned a-priori nor conducted. Secondary and exploratory analyses were performed without correction for multiple testing due to the exploratory character of these analyses.

Categorical variables were reported using absolute and relative frequencies. Continuous variables were analyzed by mean and standard deviation.

In the study protocol, analyses using multiple imputed datasets were defined for the primary outcome, and conducting analyses of complete cases (completer analyses) were planned for sensitivity analyses. Due to the high assessment drop-out in all study arms, multiple imputation seemed no longer suitable for the primary analysis. Methodological considerations regarding the mechanisms of missingness and methodological results as e.g. presented by White and Carlin (63) supported that complete case analysis is an acceptable approach in this scenario. Especially when the probability for missing values did not depend on the outcome measure itself, complete cases analysis was less biased than multiple imputation analysis (63).

Instead, we conducted completer analyses for the primary outcome and performed sensitivity analyses using the imputed datasets. This change in the analysis strategy was already pre-specified in the first version of the statistical analysis plan, signed before data analysis. No outcome data was analyzed to make this decision, only drop-out rates were considered.

The study protocol determined a non-parametric analysis (two-sided Wilcoxon rank test) to compare pre-post WCS scores within each study arm. The review of the collected data revealed a normal distribution of the score differences. Thus, the primary analysis was performed using one-sample t-tests without impairment of the validity and consistency of results and increasing power at the same time. For each study arm the primary null hypothesis $H_{0}:\;\mu_{Post}^{WCS}-\;\mu_{Pre}^{WCS}=0$ was tested.

Secondary outcomes were also analyzed using completer data. Two-sided one-sample t-tests were conducted for WCS, EDE-Q, IES, RSE, and AQoL-8D. Changes of binary outcomes, i.e. ED core symptoms (e.g. loss of control eating, excessive exercise) between assessments were analyzed using McNemar’s tests. Changes in frequency of fruit and vegetable intake were analyzed on item level using Wilcoxon signed-rank tests.

Results of t-tests were reported with effect sizes (Cohen’s *d*) for the primary and secondary outcomes including point-wise 95% confidence intervals. As descriptive statistics for the binary outcomes we reported the proportion of participants with improvements (N_improved_) at each time point among all symptomatic participants (N_symptomatic_) at baseline.

Multiple imputation for missing data was conducted via predictive mean matching, following the fully conditional specification (FCS) approach (64, 65). Due to computational issues study arm AN, data was imputed using the Markov chain Monte Carlo (MCMC) method. Separate imputation models were estimated for each study arm with m = 50 imputations. Only WCS time series data was imputed. Each time point was imputed based on statistical regression model incorporating only data from preceding time points. After imputation, pre-post within-subject differences were calculated and tested with one-sample t-tests. The single test statistics were pooled according to Rubin’s rule (66) and final test statistic and p-value was calculated.

Box plots were used to visualize median scores, mean scores, quartiles, minima, maxima and outliers of primary and secondary outcomes across time points and study arms. Measures of adherence/engagement were reported descriptively. Adverse events (yes/no) were described by absolute and relative frequencies for each type of event.

Simple linear regression models of completer data were used to assess the impact of adherence on the primary outcome, using the number of completed sessions as independent variable and WCS scores at post, 6- and 12-months follow up as dependent variables.

## 3 Results

### 3.1 Sample characteristics

**Figure 2:**
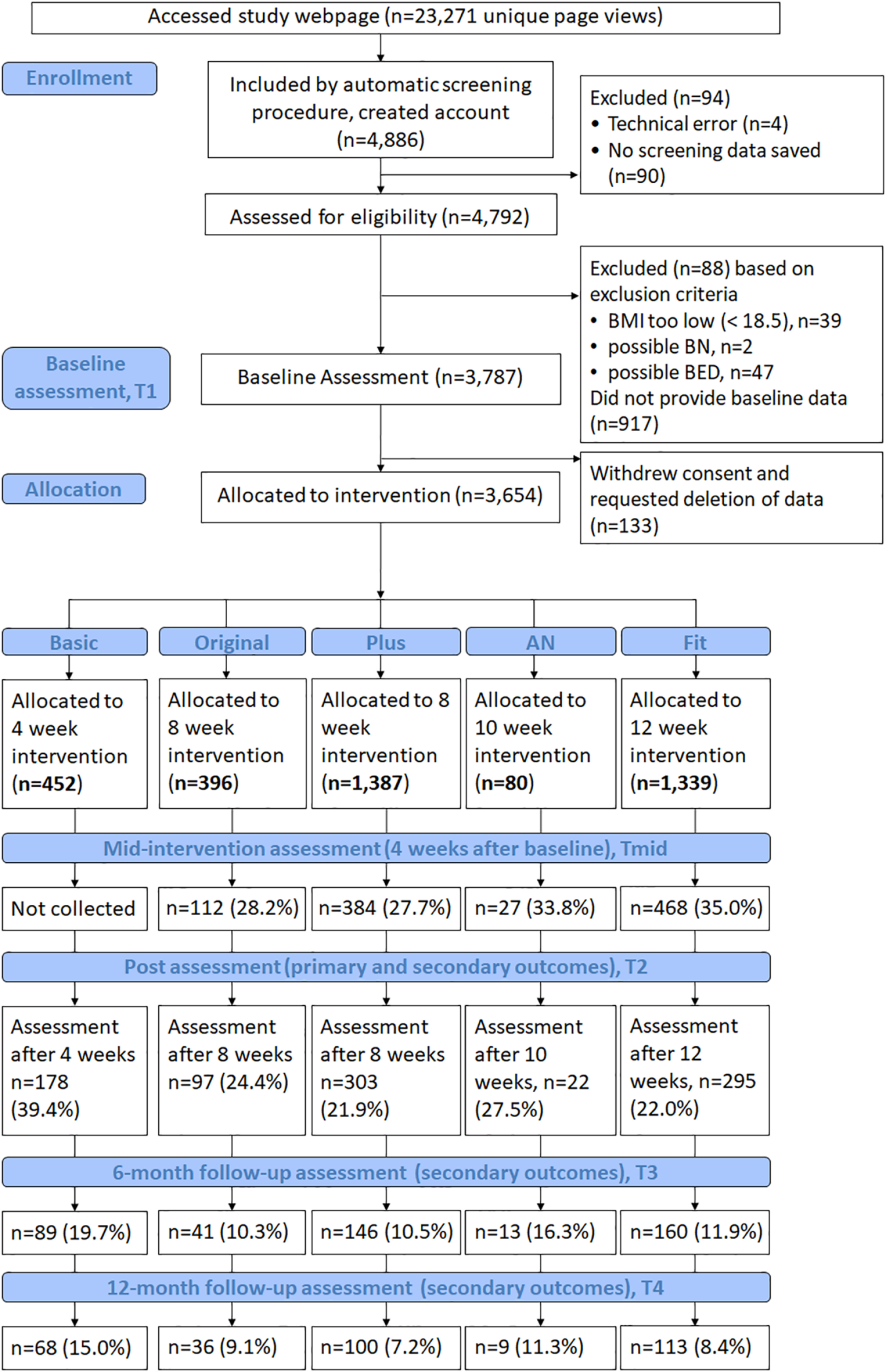
Flow of participants from opening the study website to 12-months follow-up assessments.

In total, 4,886 women were screened, 3,787 provided data for the baseline assessment and 3,654 were allocated to one of the five study arms. Of those, 452 were allocated to the study arm Basic, 396 to Original, 1,387 to Plus, 80 to AN, and 1,339 to study arm Fit. **Figure 2** presents the number of reached, screened, included, allocated and assessed participants in total and per study arm. Group sizes are based on whether any data was collected during the respective assessments, regardless of the collection of the primary outcome. Therefore, it is possible that the sample size for a specific study arm and time point is greater that the number of collected WCS (primary outcome) data.

**Table 2** presents the sociodemographic characteristics of participants at baseline. Mean age of the overall sample was 40.97 years (*SD* = 13.73, range 18 – 84). The majority of participants (66.3% to 91.3%) in each study arm had an education level of upper secondary education (European Qualifications Framework Level 4) or higher. About three quarter of the sample (72.5% to 78.9%) were in a relationship at baseline. The majority of participants were in part- or full-time employment (57.5% to 71.2%) and 8.1% to 45.0% were in training at the time. Between 22.6% (study arm Basic) and 43.8% (AN) reported a lifetime diagnosis of any mental disorder (see **Supplementary Table S1** in the appendix). Baseline fruit and vegetable intake of participants are presented in **Supplementary Table S1** in the appendix.

**Table 2:** Participants’ characteristics by study arm.

	Study arm				
	Basic	Original	Plus	AN	Fit
Variable	N = 452	N = 396	N = 1,387	N = 80	N = 1,339
Age, *M* (*SD*)	37.57 (13.45)	37.48 (13.17)	38.94 (13.71)	31.98 (11.16)	45.79 (12.67)
Age, range	18-75	18-79	18-84	18-61	18-83
BMI, *M* (*SD*)	21.83 (1.94)	22.83 (1.51)	27.65 (5.51)	19.98 (0.74)	30.54 (5.12)
Marital status, *n* (%)					
Single	111 (24.56)	89 (22.47)	379 (27.33)	22 (27.50)	283 (21.14)
In a relationship	159 (35.18)	149 (37.63)	450 (32.44)	37 (46.25)	290 (21.66)
Married, in a registered civil partnership, or shared household relationship	182 (40.27)	158 (39.90)	558 (40.23)	21 (26.25)	766 (57.21)
Occupation, *n* (%)					
Currently in training (in school, vocational training, college, or university)	122 (26.99)	101 (25.51)	326 (23.50)	36 (45.00)	109 (8.14)
Seeking employment	10 (2.21)	23 (5.81)	65 (4.69)	2 (2.50)	53 (3.96)
Housewife	19 (4.20)	19 (4.80)	98 (7.07)	4 (5.00)	113 (8.44)
Self-employed	33 (7.30)	35 (8.84)	77 (5.55)	5 (6.25)	94 (7.02)
Part time employment (less than 35 hours)	152 (33.63)	111 (28.03)	379 (27.33)	15 (18.75)	433 (32.34)
Full time employment (35 or more hours)	147 (32.52)	143 (36.02)	536 (38.67)	31 (38.75)	520 (38.83)
Retired	22 (4.87)	16 (4.04)	70 (5.05)	3 (3.75)	136 (10.16)
PHQ-9, *M* (*SD*)	5.49 (3.96)	7.34 (4.35)	9.53 (5.03)	8.51 (5.22)	6.49 (4.50)
GAD-7, *M* (*SD*)	5.15 (4.07)	6.00 (3.98)	7.43 (4.56)	6.88 (4.54)	5.23 (4.08)

PHQ-9, 9-item depression scale of the Patient Health Questionnaire; GAD-7, Generalized Anxiety Disorder 7-scale of the Patient Health Questionnaire.

### 3.2 Protocol violations

Twenty-three participants were included in the study by the screening algorithm although their BMI met the exclusion criterion of a BMI less than 18.5 (AN = 1; Basic = 21; Original = 1). Additionally, four included participants violated the inclusion criteria of the Plus program, as reasons for exclusion only became apparent during baseline or later assessments. Following the ITT principle all participants with protocol violations are analyzed in the completer as well as in the multiple imputation analysis.

### 3.3 Adherence

Participants completed an average of 2.31 (*SD* = 1.69; study arm Basic), 3.31 (*SD* = 3.13; Original), 2.68 (*SD* = 2.98; Plus), 3.70 (*SD* = 3.95; AN), and 4.55 (*SD* = 4.63; Fit) sessions. Between 44.0% (study arm Basic) and 18.0% (study arm Plus) of participants completed all respective sessions, and between 20.1% (study arm Fit) and 32.5% (study arm AN) did not complete the first session. **Supplementary Tables S2–S4** in the appendix describe adherence and engagement measures regarding session completion, platform and intervention use, use of diaries, discussion groups and messages to moderators. **Supplementary Figure S1** in the appendix illustrates the percentage of completed sessions in each study arm.

### 3.4 Outcomes

#### 3.4.1 Weight and shape concerns

For the primary outcome, there were significant within-subjects differences for the completer sample on the Weight Concerns Scale (WCS) scores from baseline to post-assessment for study arms Original, Plus, AN and Fit, with effect sizes of *d* = -0.45, 95% CI [-0.66, -0.24], *d* = -0.87 [-1.01, -0.74], *d* = -0.94 [-1.44, -0.43], and *d* = -0.54 [-0.67, -0.42], respectively (see **Table 3**). Sensitivity analyses using multiple imputation (**Supplementary Tables S11-S15** in the appendix) revealed consistent results, i.e., significant changes of WCS scores from baseline to post-assessment in the same four study arms. In the study arm Basic, significant changes in WCS scores from baseline to post-assessment were shown in the multiple imputation analysis, *d* = -0.16, 95% CI [-0.23, -0.08], but not in the completer sample.

**Table 3:** Means, standard deviations, p-value of t-tests for WCS scores, and within-subjects effect sizes from baseline to mid, post and follow-up assessments per study arm based on completer data.

	Baseline	Mid-intervention			Post			6-months follow-up			12-months follow-up		
Study arm	*M* (*SD*), *N*	*M* (*SD*), *N*	*p*	Cohen’s *d* (95% CI)	*M* (*SD*), *N*	*p*	Cohen’s *d* (95% CI)	*M* (*SD*), *N*	*p*	Cohen’s *d* (95% CI)	*M* (*SD*), *N*	*p*	Cohen’s *d* (95% CI)
Basic	29.09 (10.25), N=452	[not assessed]			27.44 (12.47), N=178	.07	-0.14 (-0.28, 0.01)	27.61 (15.76), n = 88	.46	-0.08 (-0.29, 0.13)	29.13 (16.67), n = 65	.96	-0.01 (-0.25, 0.24)
Original	51.12 (15.70), N=396	47.16 (17.58), n = 110	.11	-0.15 (-0.34, 0.04)	42.66 (16.84), N=97	<.001	-0.45 (-0.66, -0.24)	47.15 (16.45), n = 41	.18	-0.22 (-0.52, 0.10)	46.25 (18.08), n = 36	.050	-0.34 (-0.67, 0.00)
Plus	61.90 (13.35), N=1387	56.54 (15.59), n = 376	<.001	-0.52 (-0.63, -0.41)	49.23 (16.64), N=301	<.001	-0.87 (-1.01, -0.74)	44.22 (17.07), n = 145	<.001	-1.01 (-1.21, -0.81)	42.17 (18.38), n = 100	<.001	-1.13 (-1.38, -0.87)
AN	58.85 (12.07), N=80	56.67 (14.42), n = 27	.013	-0.52 (-0.91, -0.11)	50.15 (12.57), N=22	<.001	-0.94 (-1.44, -0.43)	52.36 (19.14), n = 12	.016	-0.82 (-1.46, -0.15)	52.59 (26.35), n = 9	.234	-0.43 (-1.10, 0.27)
Fit	47.43 (16.54), N=1339	43.37 (14.66), n = 453	<.001	-0.28 (-0.37, -0.18)	38.71 (15.16), N=294	<.001	-0.54 (-0.67, -0.42)	36.75 (15.65), n = 160	<.001	-0.70 (-0.88, -0.53)	35.75 (15.22), n = 111	<.001	-0.68 (-0.89, -0.47)

Negative differences and effect sizes indicate improvement in weight and shape concerns. All p-values represent comparisons with baseline.

WCS, Weight and Shape Concerns Scale.

**Figure 3:**
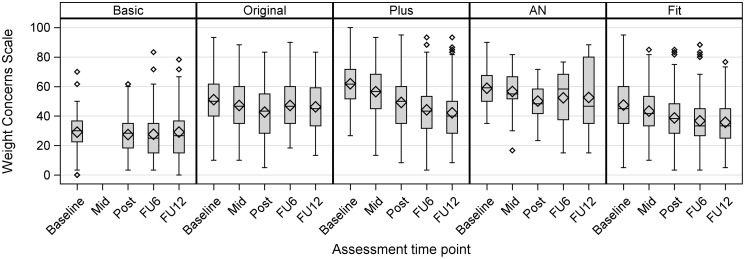
Box plots of WCS scores for each time point and study arm, depicting median scores, mean scores (diamond shape), quartiles, minima, maxima and outliers (completer data).

**Figure 3** provides an overview of WCS scores for all time points. Secondary outcomes regarding the WCS, i.e., comparisons of baseline scores with mid, post, 6-months follow-up and 12-months follow-up revealed maintained improvements of WCS scores over all time point in study arms Plus and Fit, which were confirmed by multiple imputation analyses. In study arm Original, completer data showed improvements only at post, but multiple imputation analyses show significant improvements at every time point. There were no significant within-subject differences of WCS scores in the Basic study arm except for post assessment in the multiple imputation analysis. While completer data showed significant improvements for mid assessment, post, and 6-months follow-up in study arm AN, multiple imputation analyses confirmed this effect only for the post assessment. Further sensitivity analyses using mixed model analyses confirmed the results of the primary analysis of baseline-post differences in each study arm and for most other time points as well (see **Supplementary Table S16** in the appendix).

Since no protocol violation was present in study arm Fit, the per-protocol analysis was only performed for AN, Plus, Basic and Original. The results were consistent with the completer analysis, except for Study arm Basic. In this arm WCS scores significantly improved at post-assessment (*d* = -0.16; 95% CI: -0.31, -0.01) (see **Supplementary Table S5** in the appendix).

#### 3.4.2 Secondary outcomes and follow ups

Results of the secondary outcome analyses for the completer sample are shown in **Supplementary Tables S6–S10** in the appendix. Descriptive characteristics are shown in **Supplementary Figures S2–S5** in the appendix. In study arms Plus and Fit, t-tests revealed significant improvements in EDE-Q, IES, RSE, and AQOL-8D scores between baseline and all subsequent time points. In study arms Basic, Original, and AN, scores significantly improved on most of these measures at most time points. According to the McNemar’s tests, the presence of ED symptoms in the Plus arm declined significantly for most symptoms and time points, while in the other arms, the prevalence of most core symptoms did not change significantly between time points, or McNemar’s tests could not be carried out due to too few cases. Effect sizes in the Basic study arm ranged between *d* = -0.23, 95% CI [-0.54, -0.24] for ED pathology (EDE-Q total scores; 6-month follow-up) and *d* = 0.78 [0.49, 1.07] for intuitive eating scores (IES; 12-month-follow-up), indicating improvements in these measures. In the study arm Original, effect sizes ranged between *d* = -0.35 [-0.69, -0.01] for EDE-Q scores (12-month follow-up) and *d* = -0.68 [-0.90, -0.46] for EDE-Q scores (post). Effect sizes in the Plus study arm ranged from *d* = 0.31 [0.21, 0.42] for self-esteem (RSE scores; mid-intervention) to *d* = -1.16 [-1.31, -1.02] for EDE-Q scores (post). In the AN study arm, effects ranged between *d* = 0.46 [0.01, 0.89] for IES scores (post) and *d* = -1.26 [-2.01, -0.47] for EDE-Q scores (6 month-follow up). In the Fit study arm, effects ranged between *d* = 0.20 [0.11, 0.30] for RSE scores (mid-intervention) and *d* = 0.90 [0.66, 1.13] for IES scores (12-month follow-up). Almost all significant within-subject differences in the completer sample were confirmed by multiple imputation analyses (see **Supplementary Tables S11–S15** in the appendix), except in study arm AN for IES scores at post and RSE scores at 6-months follow-up. Multiple imputation analyses additionally revealed improvements in study arm Basic (RSE at post and 12-months follow-up) and in study arm Original (EDE-Q at 6-months follow-up, IES at 12-months follow-up, RSE at 12-months follow-up). Further sensitivity analyses using mixed model analyses largely confirmed the results of the completer analyses, with few deviations (see **Supplementary Table S16** in the appendix).

#### 3.4.3 Adherence and outcomes

Analyses of linear regression models using completer data revealed that the number of completed sessions impacted improvements in WCS scores in each study arm at varying time points. In study arm Basic, participants’ average WCS score difference from 6-months follow up to baseline decreased by 10.54 for each completed session, *p* =<.001, 95% CI [-15.78, -5.30]. In study arm Original, average differences in WCS scores decreased by 2.01 per intervention session at post, *p* =<.011 [-3.55, -0.48], in study arm Plus average differences in WCS scores decreased by 1.59 at post, *p* =<.001 [-2.41, -0.77] and in study arm AN by 2.04 at post, *p* = .004 [-3.35, -0.72]. In study arm Fit, average differences in WCS scores decreased by 0.86 at post, *p* = .007 [-1.48, -0.23], and by 2.04 at 12-months follow-up, *p* =<.001 [-3.19, -0.90]. At all other time points, no significant associations were found. Results of the linear regression models are presented in **Supplementary Tables S17–S21** in the appendix.

### 3.5 Adverse events

The proportion of participants with adverse events ranged between 0% and 25% depending on study arm and assessment time point (see **Supplementary Table S22** in appendix). In study arm Original, up to 25% of participants showed adverse events AE1 and AE2 at 6-month and at 12-month follow-up, i.e., participants without and with prior core symptoms reported onset of ED symptoms or met the diagnostic threshold of one or more ED symptoms, respectively. A BMI drop below 18.0 (adverse event 3) was most prominent in study arm AN (n = 5). Inpatient treatment for ED (adverse event 4) was only reported in study arm Plus (n = 3).

## 4 Discussion

The aim of this study was to disseminate and evaluate the effectiveness, adherence, participants’ demographics, and feasibility of a suite of tailored online interventions for ED health promotion and prevention in women of the general population, including previously underserved age groups. Four of the five study arms were targeted at women at risk for EDs based on specific or common risk factors, such as weight and shape concerns, disordered eating behaviors, and other early symptoms of ED. The fifth study arm, a universal preventive intervention (study arm Basic), targeted women without any risk factors.

Overall, uptake of the combined screening and intervention approach was high with 76% (3,654 out of 4,792 women) of screened women allocated to one of the five study arms. However, intervention and assessment drop-out turned out to be markedly higher than expected. While we had estimated an assessment baseline-post drop-out rate of 50%, actual rates ranged between 60.6% and 78.1% at post-intervention and were even higher at follow-up. Intervention adherence also varied between study arms, with the highest adherence rate occurring in the shortest intervention (study arm Basic; 40.9% full completion rate).

The analyses of outcome measures suggest a beneficial impact, although the study design does not allow for causal inferences of intervention effects. Across the four intervention groups with individuals at risk for eating disorders, we found a significant reduction in weight and shape concerns, for both completer and imputed data samples. In the universal prevention study arm Basic, a significant reduction could only be found in the imputed data sample and in the per-protocol analysis. The reduction of weight and shape concerns was maintained up to 12 months for women with subthreshold binge eating symptoms and compensatory behaviors or higher BMI at baseline (study arms Plus and Fit), and up to 6 months for women with lower BMI and restrictive eating (study arm AN). The improvements in intuitive eating and ED pathology, such as eating concerns, restrained eating and weight and shape concerns were stable at most time points in all study arms. In study arm Plus, tailored for participants with initial binge eating and/or compensatory behaviors, we found stable reductions in these initial ED symptoms. The increase of fruit and vegetable intake was maintained up to 12 months in study arm Fit (targeted at women with higher BMI), which incorporated suggestions to increase fruit and vegetable intake. As study arm Basic was tailored for women with no or very low levels of weight and shape concerns and no other risk factors, we assume this group generally did not have much room for improvement. Beyond ED symptomatology, positive and stable within-subject differences up to 12 months were found for self-esteem in study arms Plus and Fit and for quality of life in study arms Basic, Plus and Fit. Finally, while weight was not an outcome measure, we found BMI was mostly stable up to 12 months follow-ups.

We found a significant impact of adherence on weight and shape concerns in all study arms, i.e., for post assessments (study arms Original, Plus, AN, and Fit), 6-months follow-up (study arm Basic) and 12-months follow-up (study arm Fit), however not for other time points. This suggests that using more of the intervention may be beneficial for participants by showing a greater reduction weight and shape concerns with greater adherence. However, we collected proportionally more outcome data of participants who finished most or all of the intervention than of those who finished fewer sessions. All five interventions had already been proven to be efficacious, i.e., shown to reduce risk factors and symptoms for ED in previous individual randomized controlled trials (25–27) or pilot studies (24, 29). The current study design, however, allowed for a tailored preventive approach from universal to selected levels and thus the inclusion of participants with very diverse ED symptomology and previously underserved age groups not reached so far.

The stable improvement in weight and shape concerns, a major risk factor for EDs and obesity, in other ED behaviors and attitudes, in self-esteem, and in quality of life for the completer sample also suggest that this approach could be beneficial under real-world conditions. Despite high drop-out rates and intervention attrition it is important to note that a total of 750 participants (20.5%) across all study arms completed the full intervention. The value of the current broad dissemination approach therefore lies in the large scale at which the intervention can be provided. As suggested by other authors, for their public health impact increasing the reach of prevention programs might be more important than solely increasing the efficacy of interventions (33).

In addition, the approach also proved feasible. The majority of participants not included in the study dropped out before and during the baseline assessment. Only a few (1.8%) screened participants were ineligible for participation, which demonstrates the feasibility of the combined screening and allocation procedure to a tailored intervention based on participants’ risk characteristics. As screening for level of risk and intervention allocation were mostly conducted automatically without including study staff resources, this tailored, population-based approach seems both promising and economic for reaching large populations and thus the scalability required for successful eating disorder prevention (32).

Another strength of the study is the assessment of long-term outcomes 12 months after inclusion. Finally, the recruitment of a large and age-diverse sample from the general population combined with minimal exclusion criteria strengthens the external validity of this trial.

However, the study also had a number of limitations. We performed separate confirmatory analyses with respect to the primary outcome within each study arm. Nevertheless, the remainder of analyses is exploratory without further correction for multiple testing and might yield the risk for false positive findings.

Assessment drop-out of this dissemination study was higher than estimated and higher compared to previous studies including StudentBodies interventions with drop-out rates between 3% and 54% (24–27, 29) or other internet-based ED prevention programs ([weighed mean post assessment drop-out rate of 21%) (18). In the current study, this might be due to the larger number of questionnaires participants had to complete, which also included questionnaires addressing moderator and mediator variables (36) not reported in this publication. Furthermore, enrollment in the trial was conducted completely online and automated. Little effort for enrollment might result in a higher number of drop-outs later on, when participants are faced with more work than initially expected (67). The outcomes of this study therefore must be interpreted on the basis of possible sampling biases due to the high drop-out rates and high proportions of imputed data. Due to the absence of specific tests to validly discriminate between data missing-at-random and data missing-not-at-random, the appropriateness of the imputation procedure may be questionable.

Intervention drop-out was also higher compared with previous studies on the interventions (24–27) or other internet-based ED prevention programs (18), but this seems to be expected in a non-randomized dissemination trial designed to increase reach. Intervention drop-out in internet-based interventions is usually higher in more open, naturalistic settings than in controlled research settings such as RCTs (68, 69). A recruitment strategy that increases reach might recruit more participants who are less motivated, reducing uptake rates and engagement (34). Recently, the personalization of frequency, timing, content, or mode of delivery of personalized reminders has been discussed for a positive impact on adherence (18). Unfortunately, due to technical constraints we were not able to adjust or personalize e-mail reminders in the study. Future revisions of the interventions could employ user-centered design methods to refine intervention content and technical features in order to improve engagement (70).

Due to its uncontrolled design, the current trial does not allow to draw causal inferences of intervention effects, as improved outcomes might also be produced by spontaneous remission or expectancy effects. However, in this large-scale dissemination trial with recruitment in the general population, including control conditions were not feasible. For example, health insurance companies were only willing to support recruitment if all eligible participants would have access to the everyBody interventions. As a large proportion of participants (49%) were recruited via health insurances, the reach of the interventions was likely only to be achieved without control groups.

Another limitation concerns the imbalance in recruitment of different risk groups. Not unexpectedly, we were only able to recruit a relatively small number of participants with restrictive eating and lower BMI for study arm AN. In the randomized controlled trial to evaluate the efficacy of this intervention, 4,646 participants had to be screened to be finally able to randomize the required number of 168 randomized participants (3.6%) (27, 71). The under-recruitment of this study arm and – consequently - the reduced statistical power might have produced fewer significant within-subject differences compared to other study arms. For the same reasons, results in this study armed should be considered exploratory in nature. It also reflects once more the difficulty to reach this particular population for prevention purposes in general (72). Finally, reports of adverse events suggest a possible later onset of eating disorder symptoms (binge eating and/or compensatory behaviors, BMI dropping below 18.0) in study arms Original and AN at 6- and 12months follow-up. Again, the respective percentages are heavily influenced by the high drop-out rates at these time points. While worsening of symptoms and treatment options were covered in the intervention content and moderator training, future dissemination programs should plan and budget for mechanisms to detect presence and onset of clinically relevant symptoms. These could include automated notifications to staff members or follow-up information to participants to inform about later onset of symptoms and courses of action.

## 5 Conclusion

Overall, the results illustrate the potential benefit of the dissemination of tailored internet-based ED prevention at a large scale. The intervention variants address a broad spectrum of risk from no risk to high risk for EDs. This could make the implementation of the suite of interventions potentially appealing to providers. The automated screening and allocation procedure provides feasible opportunities for dissemination of the prevention programs. However, presence and onset of symptoms will still have to be monitored and addressed. User experience might need to be improved to increase adherence and engagement with the interventions for future dissemination purposes.

## Data Availability

The raw data supporting the conclusions of this article will be made available by the authors, without undue reservation.
