# Supplementary material for: Tailored online eating disorder prevention and health promotion for women: results of a dissemination trial
Source: Front Psychiatry. 2026 Jan 22;16:1731066. doi: 10.3389/fpsyt.2025.1731066 (PMC12875093; doi:10.3389/fpsyt.2025.1731066)
Supplement: Supplementary file 1 [file Table1.pdf]

Supplementary material

Supplement to:

Tailored online eating disorder prevention and health promotion for women: results of a dissemination trial

Barbara Nacke, Dennis Görlich, Ina Beintner, Bianka Vollert, Juliane-Schmidt-Hantke, C. Barr Taylor, Corinna Jacobi

Corresponding Author:

Barbara Nacke
Institute of Clinical Psychology and Psychotherapy, Faculty of Psychology
Technische Universität Dresden
Germany
Email: barbara.nacke@tu-dresden.de

Table of contents

Sample characteristics 2
Adherence and Engagement 3
Per-protocol analysis of the primary outcome 6
Primary and secondary outcomes study arm Basic (completer sample) 7
Primary and secondary outcomes study arm Original (completer sample) 9
Primary and secondary outcomes study arm Plus (completer sample) 11
Primary and secondary outcomes study arm AN (completer sample) 13
Primary and secondary outcomes study arm Fit (completer sample) 15
Secondary outcomes (figures) 17
Sensitivity analyses using multiple imputed datasets 19
Sensitivity analyses using mixed model analyses 24
Relationship of adherence and outcomes 26
Adverse events 29

Sample characteristics

Table S1

Absolute and relative frequencies of self-reported lifetime diagnoses and baseline fruit and vegetable intake (consumed portions in the past 7 days) of participants by study arm

	Study arm				
	**Basic**	**Original**	**Plus**	**AN**	**Fit**
**Variable**	***n* (%)**	***n* (%)**	***n* (%)**	***n* (%)**	***n* (%)**
Lifetime diagnosis, any mental disorder	102 (22.57)	116 (29.29)	494 (35.69)	35 (43.75)	368 (27.48)
Fruit portions					
None	16 (3.54)	22 (5.56)	80 (5.77)	8 (10.00)	110 (8.22)
1 - 3 portions	126 (27.88)	79 (19.95)	321 (23.14)	21 (26.25)	335 (25.02)
4 - 6 portions	65 (14.38)	64 (16.16)	202 (14.56)	7 (8.75)	178 (13.29)
1 per day	132 (29.20)	98 (24.75)	363 (26.17)	18 (22.50)	384 (28.68)
2 per day	81 (17.92)	89 (22.42)	291 (20.98)	17 (21.25)	240 (17.92)
3 per day	23 (5.09)	33 (8.33)	92 (6.63)	7 (8.75)	70 (5.23)
4 or more per day	9 (1.99)	11 (2.78)	38 (2.74)	2 (2.50)	22 (1.64)
Vegetable portions (fresh)					
None	12 (2.65)	8 (2.02)	47 (3.39)	1 (1.25)	45 (3.36)
1 - 3 portions	115 (25.44)	78 (19.70)	302 (21.77)	12 (15.00)	338 (25.24)
4 - 6 portions	77 (17.04)	60 (15.15)	240 (17.30)	16 (20.00)	251 (18.75)
1 per day	113 (25.00)	108 (27.27)	355 (25.59)	15 (18.75)	375 (28.01)
2 per day	87 (19.25)	80 (20.20)	265 (19.11)	20 (25.00)	237 (17.70)
3 per day	33 (7.30)	40 (10.10)	127 (9.16)	15 (18.75)	65 (4.85)
4 or more per day	15 (3.32)	22 (5.56)	51 (3.68)	1 (1.25)	28 (2.09)
Vegetable portions (frozen)					
None	224 (49.56)	188 (47.47)	617 (44.48)	45 (56.25)	597 (44.59)
1 - 3 portions	173 (38.27)	151 (38.13)	530 (38.21)	22 (27.50)	544 (40.63)
4 - 6 portions	25 (5.53)	31 (7.83)	105 (7.57)	7 (8.75)	89 (6.65)
1 per day	25 (5.53)	22 (5.56)	105 (7.57)	5 (6.25)	96 (7.17)
2 per day	2 (0.44)	3 (0.76)	20 (1.44)	0 (0.00)	9 (0.67)
3 per day	2 (0.44)	1 (0.25)	4 (0.29)	1 (1.25)	2 (0.15)
4 or more per day	1 (0.22)	0 (0.00)	6 (0.43)	0 (0.00)	2 (0.15)
Smoothies, juice					
None	149 (32.96)	186 (46.72)	628 (45.28)	40 (50.00)	574 (42.87)
1 - 3 portions	193 (42.70)	135 (34.09)	490 (35.33)	25 (31.25)	480 (35.85)
4 - 6 portions	44 (9.73)	29 (7.32)	107 (7.71)	7 (8.75)	96 (7.17)
1 per day	49 (10.84)	33 (8.33)	115 (8.29)	6 (7.50)	127 (9.48)
2 per day	10 (2.21)	12 (3.03)	31 (2.24)	1 (1.25)	41 (3.06)
3 per day	4 (0.88)	1 (0.25)	11 (0.79)	1 (1.25)	13 (0.90)
4 or more per day	3 (0.66)	1 (0.25)	5 (0.36)	0 (0.00)	9 (0.67)

Adherence and Engagement

Figure S1

Intervention adherence: Percentage of participants per session who completed the session in each study arm

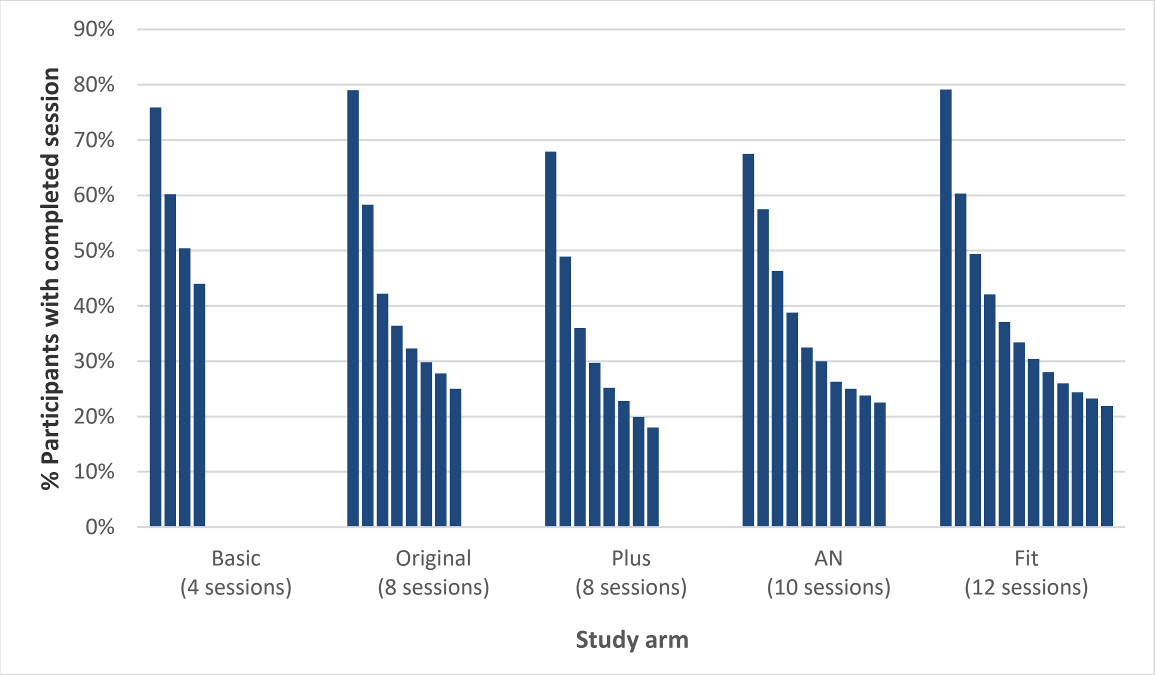

Table S2

Intervention adherence: Session completion rates for each study arm

	Study arm				
**Adherence measure**	**Basic 4 sessions** *N* = 452	**Original 8 sessions** *N* = 396	**Plus 8 sessions** *N* = 1,387	**AN 10 sessions** *N* = 80	**Fit 12 sessions** *N* = 1,339
Number of participants who completed each session, *n* (*%*)					
Session 1	343 (75.9)	313 (79.0)	942 (67.9)	54 (67.5)	1059 (79.1)
Session 2	272 (60.2)	231 (58.3)	678 (48.9)	46 (57.5)	807 (60.3)
Session 3	228 (50.4)	167 (42.2)	499 (36.0)	37 (46.3)	661 (49.4)
Session 4	199 (44.0)	144 (36.4)	412 (29.7)	31 (38.8)	563 (42.1)
Session 5	-	128 (32.3)	350 (25.2)	26 (32.5)	497 (37.1)
Session 6	-	118 (29.8)	316 (22.8)	24 (30.0)	447 (33.4)
Session 7	-	110 (27.8)	276 (19.9)	21 (26.3)	407 (30.4)
Session 8	-	99 (25.0)	250 (18.0)	20 (25.0)	375 (28.0)
Session 9	-	-	-	19 (23.8)	348 (26.0)
Session 10	-	-	-	18 (22.5)	327 (24.4)
Session 11	-	-	-	-	310 (23.2)
Session 12	-	-	-	-	293 (21.9)
Participants who completed zero sessions	109 (24.1)	83 (21.0)	445 (32.1)	26 (32.5)	280 (20.1)
Number of completed sessions					
Mean (*SD*)	2.31 (1.69)	3.31 (3.13)	2.68 (2.98)	3.70 (3.95)	4.55 (4.63)
Median (Q1; Q3)	3 (1; 4)	2 (1; 7.5)	1 (0; 5)	2 (0; 7.5)	2 (1; 9)

Table S3

Measures of platform usage per study arm

	Study arm				
**Adherence measure**	**Basic** *N* = 448[a]	**Original** *N* = 395[a]	**Plus** *N* = 1,370[a]	**AN** *N* = 78[a]	**Fit** *N* = 1,328[a]
Never logged in after baseline assessment, *n* (*%*)	62 (13.8)	33 (8.4)	139 (10.1)	9 (11.5)	103 (7.8)
Logged in at least once, *n* (*%*)	386 (86.2)	361 (91.6)	1232 (89.9)	69 (88.5)	1225 (92.2)

Note. [a] Incomplete logfile coverage for *N* = 35 participants (Basic: 4; Original: 2; Plus: 16; AN: 2; Fit: 11). To prevent bias, these participants were excluded from the logfile based platform usage analysis.

Table S4

Participant's use of interactive intervention components (diaries, discussion groups and moderator support)

	Study arm				
	**Basic**	**Original**	**Plus**	**AN**	**Fit**

**Adherence measure**	*N* = 386[a]	*N* = 361[a]	*N* = 1,232[a]	*N* = 69[a]	*N* = 1,225[a]
Entries in sessions (free text fields; percentage based on number of maximum possible entries)					
Median (Q1; Q3) %	n.a.	n.a.	70% (55%; 85%)	n.a.	64% (50%; 83%)
Mean (SD) %	n.a	n.a	68 (23)	n.a	64 (22)
Number of participants who filled in (at least one) diary, *n* (*%*)	n.a.	107 (27.2)	1097 (79.1)	59 (73.8)	1009 (75.4)
Number of participants who posted in discussion group, n (%)	n.a.	64 (17.6)	246 (19.8)	16 (22.5)	328 (26.6)
Of those: Number of messages in group discussion					
Mean (*SD*)	n.a.	5.42 (8.84)	8.70 (22.36)	1.63 (0.89)	6.55 (11.56)
Median (Q1;Q3)	n.a.	3 (1; 5.5)	3 (1; 7)	1 (1; 2)	3 (1; 7)
Range	n.a.	1 - 63	1 - 241	1 - 4	1 - 110
Number of participants who sent (at least one) message to moderator, n (%)	44 (11.3)	31 (8.5)	602 (48.4)	38 (53.5)	196 (16.0)
Of those: Number of direct messages to moderator					
Mean (SD)	1.61 (1.15)	1.42 (0.62)	3.01 (2.65)	3.71 (2.77)	2.08 (2.52)
Median (Q1;Q3)	1 (1;2)	1 (1;2)	2 (1;4)	3 (1;5)	1 (1;2)
Range	1 - 7	0 - 3	1 - 20	1 - 10	1 - 30

Note. [a] Group size (*N*) refers to participants who logged in at least once on intervention platform.

Per-protocol analysis of the primary outcome

Table S5

Means, standard deviations, results of t-tests for WCS scores (primary outcome), within-subjects effect sizes from baseline to post assessment per study arm based on the per-protocol completer data

**Study arm**	**Baseline** ***M*** **(*SD*), *N***	**Post** ***M*** **(*SD*), *N***	**Mean baseline-post differences (*SD*)**	***t*** **(*df*)**	***P***	**Cohen's *d* (95% CI)**
Basic	29.42 (9.37) N=431	27.48 (12.23) N=164	Δ = -1.50 (9.34)	-2.06 (163)	.041	-0.16 (-0.31, -0.01)
Original	51.85 (15.31) N=395	42.88 (16.80) N=96	Δ = -6.33 (13.86)	-4.48 (95)	<.001	-0.46 (-0.67, -0.25)
Plus	62.24 (12.88) N=1383	49.19 (16.69) N=299	Δ = -12.94 (14.81)	-15.11 (298)	<.001	-0.87 (-1.01, -0.74)
AN	60.38 (10.63) N=79	50.15 (12.57) N=22	Δ = -9.92 (10.54)	-4.41 (21)	<.001	-0.94 (-1.44, -0.43)

Primary and secondary outcomes study arm Basic (completer sample)

Table S6

Secondary outcomes in study arm Basic (completer sample)

**Baseline**	**Mid-intervention**			**Post**				**6-months follow-up**				**12-months follow-up**			
**n, mean (SD)**	**Mean (SD)**	**Cohen's *d* (95%CI)**	***t* statistic (*df*)**	**Mean (SD)**	**Cohen's *d* (95% CI)**	***t* statistic (*df*)**	***p***	**Mean (SD)**	**Cohen's *d* (95% CI)**	***t* statistic (*df*)**	***p***	**Mean (SD)**	**Cohen's *d* (95% CI)**	***t* statistic (*df*)**	***p***
Weight Concerns (WCS, 0-100)															
n = 452<br>29.09<br>(10.25)	-	-	-	n = 178<br>27.44<br>(12.47)	-0.14 (-0.28, 0.01)	-1.82<br>(177)	.07	n = 88<br>27.61<br>(15.76)	-0.08 (-0.29, 0.13)	-0.74 (87)	.464	n = 65<br>29.13<br>(16.67)	-0.01 (-0.25, 0.24)	-0.05<br>(64)	.96
Eating Disorder Examination Questionnaire total score (EDE-Q, 0-6)															
n = 452<br>1.18 (0.67)	-	-	-	n = 177<br>0.95 (0.68)	-0.39<br>(-0.54, -0.24)	-5.22<br>(176)	<.001	n = 88<br>0.98 (0.92)	-0.23<br>(-0.44, -0.02)	-2.14 (87)	.035	n = 65<br>0.97 (0.83)	-0.38<br>(-0.63, -0.13)	-3.08<br>(64)	.003
Intuitive Eating Scale (IES, 1-5)															
n = 452<br>3.55 (0.55)				n = 166<br>3.70 (0.57)	0.44 (0.28, 0.60)	5.63<br>(165)	<.001	n = 80<br>3.87 (0.62)	0.75 (0.49, 0.99)	6.68 (79)	<0.001	n = 59<br>3.79 (0.62)	0.78 (0.49, 1.07)	5.98 (58)	<.001
Rosenberg Self-Esteem Scale, total score (RSE, 10-40)															
n = 452<br>32.49<br>(5.32)				n = 166<br>33.49<br>(5.27)	0.12 (-0.04, 0.27)	1.51<br>(165)	.1319	n =81<br>33.46<br>(5.60)	0.27 (0.05, 0.49)	2.42 (80)	.0179	n = 60<br>33.72<br>(4.85)	0.26 (0.00, 0.51)	2.00 (59)	.050
Assessment of Quality of Life-8D, Utility score (AQoL-8D, 1-5)															
n = 386<br>0.72 (0.15)				n = 166<br>0.75 (0.15)	0.32 (0.16, 0.48)	4.05<br>(157)	<.001	n = 81<br>0.76 (0.17)	0.37 (0.14, 0.59)	3.26 (78)	.002	n = 59<br>0.77 (0.15)	0.42 (0.15, 0.69)	3.24 (57)	.002
BMI															
n = 452<br>21.85<br>(1.94)				n = 177<br>21.77<br>(2.20)	-.01 (-0.15, 0.14)	-0.09<br>(176)	.925	n = 86<br>21.53<br>(2.30)	0.0<br>(-0.21, 0.21)	-0.0 (85)	.996	n = 63<br>22.08<br>(2.45)	0.01<br>(-0.24,0.25)	0.04 (62)	0.967
**Presence of ED Core symptoms (yes vs. no), past 4 weeks**															
n = 452				n = 177				n = 86				n = 63			
**N, % with symptom present**				**n, % with symptom present**	**$N_{improved}$ / $N_{symptomatic}$ (%)**	**χ2 (*df*)**	***p***	**n, % with symptom present**	**$N_{improved}$ / $N_{symptomatic}$ (%)**	**χ2 (*df*)**	***p***	**n, % with symptom present**	**$N_{improved}$ / $N_{symptomatic}$ (%)**	**χ2 (*df*)**	***p***
Eating large amounts of food															
n = 23<br>5.1%				n = 10<br>5.7%	5/10 (50)	0.00 (1)	1.00	n = 8<br>9.3%	4/4 (100)	1.33 (1)	.25	n = 9<br>14.3%	3/4 (75)	2.27 (1)	.13
Loss of control eating															
n = 37<br>8.2%				n = 14<br>7.9%	8/12 (41.7)	0.06 (1)	.81	n = 10<br>11.6%	5/9 (55.6)	0.09 (1)	.76	n = 7<br>11.1%	6/8 (75)	0.09 (1)	.76

Baseline	Mid-intervention	Post				6-months follow-up				12-months follow-up			
Excessive exercise													
n = 22<br>4.9%		n = 5<br>2.8%	6/10 (60)	3.57 (1)	.06	n = 3<br>3.5%	3/4 (75)	0.20 (1)	.65	n = 3<br>4.8 %	1/3 (33.3)	0.00 (1)	1.00
Fasting to maintain weight/figure													
n = 39<br>8.6%		n = 11<br>6.2%	9/13 (69.2)	0.25 (1)	.06	n = 7<br>8.1%	4/7	0.00 (1)	1.00	n = 8<br>12.7%	3/5 (60)	1.00 (1)	.31
Appetite suppressant													
n = 0		n = 0	[no cases at baseline]			n = 0	[no cases at baseline]			n = 0	[no cases at baseline]		
Use of diuretics													
n = 1<br>0.2%		n = 0<br>0.0%	0/0	[no cases]		n = 0<br>0.0%	0/0	[no cases]		n = 0<br>0.0%	0/0	[no cases]	
Use of laxatives													
n = 0<br>0.0%		n = 0<br>0.0%	[no cases at baseline]	[no cases]		n = 1<br>1.2%	[no cases at baseline]	[no cases at baseline]		n = 0<br>0.0%	[no cases at baseline]	[no cases]	
Induced vomiting													
n = 2<br>0.4%		n = 1<br>0.6%	2/2 (100)	0.33 (1)	.56	n = 2<br>2.3%	2/2 (100)	0.00 (1)	1.00	n = 0<br>0.0%	1/1 (100)	[no cases]	
**Fruit and vegetable intake** (range: 0-6; 0 in past 7 days, 4 or more portions per day)													
n = 452			n = 166				n = 80				n = 58		
**Median (Q1; Q3)**		**Median (Q1; Q3)**	**Median difference, mean difference**	**S**	***p***	**Median (Q1; Q3)**	**Median difference, mean difference**	**S**	***p***	**Median (Q1; Q3)**	**Median difference, mean difference**	**S**	***p***
Fresh fruit													
3 (1; 3.5)		3 (2; 4)	0 (median)<br>0.10 (mean)	220	.35	3 (2; 4)	0 (median)<br>0.35 (mean)	254	.005	3 (1; 4)	0 (median)<br>0.09 (mean)	32.5	.53
Vegetables (fresh)													
3 (1; 4)		3 (2; 4)	0 (median)<br>0.12 (mean)	553.5	.020	3 (2; 4)	0 (median)<br>0.31 (mean)	192.5	.034	3 (2; 4)	0 (median)<br>0.05 (mean)	29	.69
Vegetables (frozen)													
1 (0; 1)		1 (0; 1)	0 (median)<br>0.04 (mean)	63.5	.71	0 (0; 1)	0 (median)<br>0.09 (mean)	46	.46	1 (0; 1)	0 (median)<br>0.10 (mean)	31	.44
Smoothies/juice													
1 (0; 1)		1 (0; 2)	0 (median)<br>-0.01 (mean)	-86.5	.67	1 (0; 2)	0 (median)<br>0.14 (mean)	84	.26	1 (0; 1)	0 (median)<br>0 (mean)	1.5	.97

Note. In study arm Basic no mid-intervention assessments were conducted. All p-values represent comparisons with baseline.

Primary and secondary outcomes study arm Original (completer sample)

Table S7

Secondary outcomes in study arm Original (completer sample)

**Baseline**	**Mid-intervention**				**Post**				**6-months follow-up**				**12-months follow-up**			
**n, mean (SD)**	**Mean (SD)**	**Cohen's *d* (95% CI)**	***t* statistic (*df*)**	***p***	**Mean (SD)**	**Cohen's *d* (95% CI)**	***t* statistic (*df*)**	***p***	**Mean (SD)**	**Cohen's *d* (95% CI)**	***t* statistic (*df*)**	***p***	**Mean (SD)**	**Cohen's *d* (95% CI)**	***t* statistic (*df*)**	***p***
Weight Concerns (WCS, 0-100)																
n = 396<br>51.12<br>(15.70)	n = 110<br>47.16<br>(17.58)	-0.15 (-0.34, 0.03)	-1.60 (109)	.113	n = 97<br>42.66<br>(16.85)	-0.45 (-0.66, -0.24)	-4.47 (96)	<.001	n = 41<br>47.15<br>(16.45)	-0.22 (-0.52, 0.10)	-1.38 (40)	.176	n = 36<br>46.25<br>(18.08)	-0.34 (-0.67, 0.00)	-2.03 (35)	.050
Eating Disorder Examination Questionnaire total score (EDE-Q, 0-6)																
n = 396<br>2.30<br>(0.97)	n = 109<br>1.90<br>(1.03)	-0.52 (-0.72, -0.32)	-5.41 (108)	<.001	n = 96<br>1.67<br>(1.03)	-0.68 (-0.90, -0.46)	-6.65 (95)	<.001	n = 41<br>1.88<br>(1.26)	-0.31 (-0.63, 0.00)	-2.01 (40)	.051	n = 36<br>2.05<br>(1.28)	-0.35 (-0.69, -0.01)	-2.11 (35)	.042
Intuitive Eating Scale (IES, 1-5)																
n = 396<br>3.03<br>(0.51)	n = 109<br>3.26<br>(0.57)	0.45 (0.25, 0.64)	4.66 (108)	<.001	n = 90<br>3.35<br>(0.65)	0.63 (0.40, 0.85)	5.94 (89)	<.001	n = 37<br>3.37<br>(0.78)	0.46 (0.12, 0.80)	2.81 (36)	.008	n = 32<br>3.25<br>(0.79)	0.32 (-0.04, 0.67)	1.79 (31)	.084
Rosenberg Self-Esteem Scale (RSE, 10-40)																
n = 396<br>29.73<br>(6.01)	n = 107<br>30.10<br>(5.66)	0.11 (-0.08, 0.30)	1.18 (106)	0.243	n = 90<br>31.76<br>(5.32)	0.43 (0.21, 0.65)	4.09 (89)	<.001	n = 39<br>30.15<br>(5.77)	0.13 (-0.19, 0.45)	0.82 (38)	.418	n = 33<br>31.03<br>(6.09)	0.23 (-0.12, 0.58)	1.33 (32)	.192
Assessment of Quality of Life-8D (AQoL-8D, 1-5)																
n = 349<br>0.68<br>(0.15)	[not assessed]				n = 90<br>0.73<br>(0.16)	0.43 (0.21, 0.64)	4.01 (87)	<.001	n = 39<br>0.68<br>(0.18)	0.04 (-0.28, 0.36)	0.23 (37)	.82	n = 33<br>0.69<br>(0.16)	0.06 (-0.29, 0.41)	0.35 (31)	.73
BMI																
n = 396<br>22.83<br>(1.57)	[not assessed]				n = 94<br>22.67<br>(1.59)	-0.22 (-0.42, -0.01)	-2.13 (94)	.036	n = 41<br>23.12<br>(2.96)	-0.10 (-0.41, 0.20)	-0.67 (40)	.507	n = 36<br>23.04<br>(3.25)	-0.18 (-0.50, 0.15)	-1.06	.298
**Presence of ED Core symptoms (yes vs. no), past 4 weeks**																
n = 396	n = 112				n = 97				n = 41				n = 36			
**N, % with symptom present**	**n, % with symptom present**	**$N_{improved}$ / $N_{symptomatic}$ (%)**	**χ2 (*df*)**	***p***	**n, % with symptom present**	**$N_{improved}$ / $N_{symptomatic}$ (%)**	**χ2 (*df*)**	***p***	**n, % with symptom present**	**$N_{improved}$ / $N_{symptomatic}$ (%)**	**χ2 (*df*)**	***p***	**n, % with symptom present**	**$N_{improved}$ / $N_{symptomatic}$ (%)**	**χ2 (*df*)**	***p***
Eating large amounts of food																
n = 55<br>13.9%	n = 15<br>13.4%	6/14 (42.9)	0.08 (1)	.78	n = 10<br>10.3%	2/9 (22.2)	0.20 (1)	.65	n = 9<br>22.0%	3/6 (50)	1.00 (1)	.32	n = 6<br>16.7%	4/5 (80)	0.11 (1)	.74
Loss of control eating																
n = 95<br>24.0%	n = 23<br>20.5%	22/34 (64.7)	3.67 (1)	.055	n = 15<br>15.5%	15/26 (57.7)	6.37 (1)	.012	n = 8<br>19.5%	7/10 (70)	0.33 (1)	.56	n = 11<br>30.6%	6/9 (66.7)	0.29 (1)	.59

**Baseline**	**Mid-intervention**				**Post**				**6-months follow-up**				**12-months follow-up**			
Excessive exercise																
n = 33<br>8.3%	n = 10<br>8.9%	5/11 (45.5)	0.11 (1)	.74	n = 8<br>8.3%	4/8 (50)	0.00 (1)	1.00	n = 3<br>7.3%	1/2 (50)	0.33 (1)	.56	n = 4<br>11.1%	2/4 (50)	0.00 (1)	1.00
Fasting to maintain weight/figure																
n = 75<br>18.9%	n = 12<br>10.7%	14/23 (60.9)	7.12 (1)	.008	n = 10<br>10.3%	14/21 (66.7)	7.12 (1)	.008	n = 3<br>7.3%	7/8 (87.5)	2.78 (1)	.10	n = 7<br>19.4%	9/12 (75)	1.92 (1)	.17
Appetite suppressants																
n = 7<br>1.8%	n = 1<br>0.9%	1/2 (50)	1.00 (1)	.32	n = 1<br>1.0%	3/3 (100)	1.00 (1)	.32	n = 1<br>2.4%	1/1 (100)	0.00 (1)	1.00	n = 0<br>0%	1/1 (100)	[no cases]	
Use of diuretics																
n = 1<br>0.3%	n = 0	0/0	[no cases]		n = 0<br>0%	0/0	[no cases]		n = 0<br>0%	0/0	[no cases]		n = 0<br>0%	0/0	[no cases]	
Use of laxatives																
n = 3<br>0.8%	n = 0<br>0%	2/2 (100)	[no cases]		n = 0<br>0%	1/1 (100)	[no cases]		n = 0<br>0%	1/1 (100)	[no cases]		n = 0<br>0%	1/1 (100)	[no cases]	
Induced vomiting																
n = 11<br>2.8%	n = 2<br>1.8%	0/1 (0)	1.00 (1)	.32	n = 1<br>1.0%	0/1 (0)	[no discordant pairs]		n = 1<br>2.4%	0/0	1.00 (1)	.32	n = 1<br>2.8%	0/1 (0)	[no discordant pairs]	
**Fruit and vegetable intake** (0-6)																
n = 396	n = 109				n = 90				n = 37				n = 32			
**Median (Q1; Q3)**	**Median (Q1; Q3)**	**Median difference, mean difference**	**S**	***p***	**Median (Q1; Q3)**	**Median difference, mean difference**	**S**	***p***	**Median (Q1; Q3)**	**Median difference, mean difference**	**S**	***p***	**Median (Q1; Q3)**	**Median difference, mean difference**	**S**	***p***
Fresh fruit																
3 (1; 4)	3 (2; 4)	0 (median)<br>0.02 (mean)	55	.64	3 (2; 4)	0 (median)<br>0.09 (mean)	78.5	.31	3 (1; 4)	0 (median)<br>-0.43 (mean)	-63	.06	3 (2; 4)	0 (median)<br>0.13 (mean)	16.5	.38
Vegetables (fresh)																
3 (2; 4)	3 (3; 4)	0 (median)<br>0.03 (mean)	26	.81	3.5 (3; 4)	0 (median)<br>0.16 (mean)	120	.19	3 (2; 4)	0 (median)<br>-0.35 (mean)	-33	.31	3 (2; 4.5)	0 (median)<br>0.06 (mean)	5	.87
Vegetables (frozen)																
1 (0; 1)	0 (0; 1)	0 (median)<br>-0.08 (mean)	-78	.47	1 (0; 1)	0 (median)<br>0.16 (mean)	130.5	.15	1 (0; 1)	0 (median)<br>0.16 (mean)	18.5	.35	1 (0; 1)	0 (median)<br>0.16 (mean)	7	.70
Smoothies/juice																
1 (0; 1)	1 (0; 1)	0 (median)<br>0.12 (mean)	153.5	.09	1 (0; 1)	0 (median)<br>0.28 (mean)	197.5	.003	1 (0; 1)	0 (median)<br>0.24 (mean)	34.5	.14	1 (0; 1.5)	0 (median)<br>0.25 (mean)	35.5	.13

Note. All p-values represent comparisons with baseline.

Primary and secondary outcomes study arm Plus (completer sample)

Table S8

Secondary outcomes in study arm Plus (completer sample)

**Baseline**	**Mid-intervention**				**Post**				**6-months follow-up**				**12-months follow-up**			
**n, mean (SD)**	**Mean (SD)**	**Cohen's *d* (95%CI)**	***t* statistic (*df*)**	***p***	**Mean (SD)**	**Cohen's *d* (95%CI)**	***t* statistic (*df*)**	***p***	**Mean (SD)**	**Cohen's *d* (95%CI)**	***t* statistic (*df*)**	***p***	**Mean (SD)**	**Cohen's *d* (95%CI)**	***t* statistic (*df*)**	***p***
Weight Concerns (WCS, 0-100)																
n = 1387 61.90 (13.34)	n = 376 56.54 (15.59)	-0.52 (-0.63, -0.41)	-10.10 (375)	<.001	n = 301 49.24 (16.65)	-0.87 (-1.01, -0.74)	-15.17 (300)	<.001	n = 145 44.22 (17.07)	-1.01 (-1.21, -0.81)	-12.14 (144)	<.001	n = 100 42.17 (18.38)	-1.13 (-1.38, -0.87)	-11.26 (99)	<.001
Eating Disorder Examination Questionnaire total score (EDE-Q, 0-6)																
n = 1387 3.19 (0.91)	n = 376 2.44 (1.03)	-0.97 (-1.09, -0.85)	-18.84 (375)	<.001	n = 301 1.97 (1.12)	-1.16 (-1.31, -1.02)	-20.21 (300)	<.001	n = 143 1.75 (1.17)	-1.16 (-1.37, -0.94)	-13.83 (142)	<.001	n = 100 1.69 (1.18)	-1.12 (-1.48, -0.96)	-12.25 (99)	<.001
Intuitive Eating Scale (IES, 1-5)																
n = 1387 2.59 (0.47)	n = 370 2.91 (0.56)	0.76 (0.65, 0.88)	14.69 (369)	<.001	n = 281 3.20 (0.60)	1.00 (0.86, 1.15)	16.82	<.001	n = 132 3.28 (0.64)	0.97 (0.76, 1.17)	11.10 (131)	<.001	n = 93 3.34 (0.67)	1.21 (0.94, 1.47)	11.64 (92)	<.001
Rosenberg Self-Esteem Scale (RSE, 10-40)																
n = 1387 28.04 (6.45)	n = 369 29.35 (6.24)	0.31 (0.21, 0.42)	6.05 (368)	<.001	n = 284 31.19 (6.12)	0.59 (0.47, 0.72)	9.98 (283)	<.001	n = 135 31.91 (6.47)	0.72 (0.53, 0.91)	8.38 (134)	<.001	n = 93 31.40 (6.13)	0.72 (0.49, 0.95)	6.93 (92)	<.001
Assessment of Quality of Life-8D (AQoL-8D, 1-5)																
n = 1205 0.61 (0.17)	[not assessed]				n = 283 0.68 (0.18)	0.58 (0.45, 0.70)	9.53 (272)	<.001	n = 134 0.69 (0.19)	0.67 (0.48, 0.86)	7.66 (130)	<.001	n = 0.69 (0.19)	0.67 (0.44, 0.90)	6.41 (90)	<.001
BMI																
n = 1387 27.64 (5.51)	[not assessed]				n = 300 27.22 (6.07)	0.00 (-0.11, 0.11)	0.05	.964	n = 142 24.01 (4.84)	-0.24 -0.41, -0.08)	-2.89 (141)	.004	n = 98 27.03 (5.12)	-.15 (-0.35, 0.05)	-1.49 (97)	.140
**Presence of ED core symptoms (yes vs. no), past 4 weeks**																
n = 1387	n = 372				n = 301				n = 143				n = 98			
**N, % with symptom present**	**n, % with symptom present**	**$N_{improved}$ / $N_{symptomatic}$ (%)**	**χ2 (*df*)**	***p***	**n, % with symptom present**	**$N_{improved}$ / $N_{symptomatic}$ (%)**	**χ2 (*df*)**	***p***	**n, % with symptom present**	**$N_{improved}$ / $N_{symptomatic}$ (%)**	**χ2 (*df*)**	***p***	**n, % with symptom present**	**$N_{improved}$ / $N_{symptomatic}$ (%)**	**χ2 (*df*)**	***p***
Eating large amounts of food																
n = 462 33.3%	n = 122 32.8%	59/126 (46.8)	0.14 (1)	.71	n = 96 31.9%	43/99 (43.4)	0.11 (1)	.74	27 18.9%	28/46 (60.9)	9.76 (1)	.002	23 23.5%	19/31 (61.3)	2.13 (1)	.14
Loss of control eating																
n = 1044 75.3%	n = 195 52.4%	115/294 (39.1)	74.82 (1)	<.001	n = 137 45.5%	118/248 (47.6)	98.57 (1)	<.001	n = 49 34.27%	65/108 (60.2)	49.03 (1)	<.001	n = 37 37.8%	46/77 (59.7)	30.77 (1)	<.001

Baseline	Mid-intervention				Post				6-months follow-up				12-months follow-up			
Excessive exercise																
n = 150<br>10.8%	n = 23<br>6.2%	35/50 (70)	16.95 (1)	<.001	n = 19<br>6.3%	28/42 (66.7)	16.03 (1)	<.001	n = 8<br>5.6%	12/15 (80)	2.88 (1)	.09	n = 8<br>8.2%	15/19 (78.9)	6.37 (1)	.012
Fasting to maintain weight/figure																
n = 337<br>24.3%	n = 61<br>6.4%	61/106 (57.6)	26.30 (1)	<.001	n = 35<br>11.6%	53/79 (67.1)	31.23 (1)	<.001	n = 24<br>16.8%	28/38 (73.7)	4.67 (1)	.031	n = 13<br>13.3%	19/26 (73.1)	6.76 (1)	.009
Appetite suppressants																
n = 41<br>3.0%	n = 9<br>2.4%	8/10 (80)	0.07 (1)	.80	n = 2<br>0.7%	9/10 (90)			n = 0<br>0.0%	3/3 (100)			n = 0<br>0.0%	2/2 (100)		
Use of diuretics																
n = 56<br>4.0%	n = 8<br>2.2%	9/15 (60)	4.45 (1)	.035	n = 4<br>1.3%	14/17 (82.4)	11.27 (1)	<.001	n = 2<br>1.4%	5/7 (71.4)	5.00 (1)	.025	n = 1<br>1.0%	5/6 (83.3)	5.00 (1)	.025
Use of laxatives																
n = 52<br>3.8%	n = 19<br>5.1%	3/13 (23.1)	3.00 (1)	.08	n = 4<br>1.3%	11/13 (84.6)	6.23 (1)	.013	n = 2<br>1.4%	7/8 (87.5)	4.50 (1)	.034	n = 2<br>2.0%	2/3 (66.7)	0.33 (1)	.56
Induced vomiting																
n = 84<br>6.1%	n = 20<br>5.4%	10/25 (40)	1.67 (1)	.20	n = 16<br>5.3%	7/18 (38.9)	0.33 (1)	.56	n = 11<br>7.7%	3/10 (30)	0.14 (1)	.71	n = 3<br>3.1%	1/3 (33.3)	0.00 (1)	1.00
**Fruit and vegetable intake** (range: 0-6)																
n = 1387	n = 370				n = 281				n = 133				n = 93			
**Median (Q1; Q3)**	**Median (Q1; Q3)**	**Median difference, mean difference**	**S**	***p***	**Median (Q1; Q3)**	**Median difference, mean difference**	**S**	***p***	**Median (Q1; Q3)**	**Median difference, mean difference**	**S**	***p***	**Median (Q1; Q3)**	**Median difference, mean difference**	**S**	***p***
Fresh fruit																
3 (1; 4)	3 (2; 4)	0 (median)<br>0.11 (mean)	1289.5	.11	3 (2; 4)	0 (median)<br>0.02 (mean)	185	.74	3 (2; 4)	0 (median)<br>0.05 (mean)	70	.73	3 (2; 4)	0 (median)<br>-0.17 (mean)	-230	.13
Vegetables (fresh)																
3 (2; 4)	3 (2; 4)	0 (median)<br>0.13 (mean)	1835.5	.033	3 (2; 4)	0 (median)<br>0.14 (mean)	1129.5	.07	3 (2; 4)	0 (median)<br>0.18 (mean)	358	.06	3 (2; 4)	0 (median)<br>0.25 (mean)	213.5	.06
Vegetables (frozen)																
0 (0; 1)	1 (0; 1)	0 (median)<br>-0.11 (mean)	-1140.5	.044	1 (0; 1)	0 (median)<br>-0.02 (mean)	-102.5	.79	1 (0; 1)	0 (median)<br>-0.12 (mean)	-143	.32	1 (0; 1)	0 (median)<br>-0.22 (mean)	-150	.12
Smoothies/juice																
1 (0; 1)	1 (0; 1)	0 (median)<br>-0.13 (mean)	-1450.5	.015	1 (0; 1)	0 (median)<br>-0.03 (mean)	-248	.48	0 (0; 1)	0 (median)<br>-0.11 (mean)	-204.5	.14	1 (0; 1)	0 (median)<br>0.02 (mean)	6	.95

Note. All p-values represent comparisons with baseline.

Primary and secondary outcomes study arm AN (completer sample)

Table S9

Secondary outcomes in study arm AN (completer sample)

**Baseline**	**Mid-intervention**				**Post**				**6-months follow-up**				**12-months follow-up**			
**n, mean (SD)**	**Mean (SD)**	**Cohen's *d* (95%CI)**	***t* statistic (*df*)**	***p***	**Mean (SD)**	**Cohen's *d* (95%CI)**	***t* statistic (*df*)**	***p***	**Mean (SD)**	**Cohen's *d* (95%CI)**	***t* statistic (*df*)**	***p***	**Mean (SD)**	**Cohen's *d* (95%CI)**	***t* statistic (*df*)**	***p***
Weight Concerns (WCS, 0-100)																
n = 80 58.85 (12.07)	n = 27 56.67 (14.42)	-0.52 (-0.91, -0.11)	-2.68 (26)	.013	n = 22 50.15 (12.57)	-0.94 (-1.44, -0.43)	-4.41 (21)	<.001	n = 12 52.36 (19.14)	-0.82 (-1.46, -0.15)	-2.84 (11)	.016	n = 9 52.59 (26.35)	-0.43 (-1.10, 0.27)	-1.29 (8)	.234
Eating Disorder Examination Questionnaire total score (EDE-Q, 0-6)																
n = 80 2.68 (1.11)	n = 27 2.31 (1.05)	-0.85 (-1.29, -0.40)	-4.41 (26)	<.001	n = 22 1.92 (1.04)	-0.95 (-1.44, -0.43)	-4.44 (21)	<.001	n = 12 2.21 (1.21)	-1.26 (-2.01, -0.47)	-4.36 (11)	.001	n = 9 2.50 (1.63)	-0.42 (-1.09, 0.28)	-1.25 (8)	.247
Intuitive Eating Scale (IES, 1-5)																
n = 80 3.10 (0.52)	n = 27 3.40 (0.59)	0.67 (0.25, 1.08)	3.48 (26)	.002	n = 22 3.50 (0.72)	0.46 (0.01, 0.89)	2.15 (21)	0.04	n = 12 3.46 (0.60)	0.71 (0.07, 1.34)	2.48 (11)	.030	n = 9 3.59 (0.66)	1.28 (0.36, 2.15)	3.83 (8)	.005
Rosenberg Self-Esteem Scale (RSE, 10-40)																
n = 80 26.80 (7.18)	n = 27 26.37 (6.30)	0.35 (-0.04, 0.73)	1.82 (26)	.08	n = 22 28.41 (7.25)	0.39 (-0.05, 0.81)	1.81 (21)	.09	n = 12 25.08 (5.82)	0.97 (0.26, 1.65)	3.35 (11)	.007	n = 9 25.67 (6.38)	0.73 (-0.03, 1.46)	2.19 (8)	.06
Assessment of Quality of Life-8D (AQoL-8D, 1-5)																
n = 71 0.64 (0.17)	[not assessed]				n = 22 0.61 (0.15)	0.32 (-0.12, 0.75)	1.46 (20)	.16	n = 12 0.56 (0.16)	0.43 (-0.18, 1.01)	1.47 (11)	.17	n = 9 0.56 (0.12)	0.23 (-0.44, 0.88)	0.68 (8)	.52
BMI																
n = 80 19.99 (0.74)	[not assessed]				n = 22 20.03 (1.08)	0.10 (-0.32, 0.51)	0.45 (21)	.656	n = 12 19.20 (1.15)	-0.63 (-1.24, 0.003)	-2.18 (11)	.051	n = 9 19.10 (1.88)	-0.36 (-1.02, 0.33)	-1.07 (8)	.317
**Presence of ED Core symptoms (yes vs. no), past 4 weeks**																
n = 80	n = 27				n = 22				n = 12				n = 9			
**N, % with symptom present**	**n, % with symptom present**	**$N_{improved}$ / $N_{symptomatic}$ (%)**	**χ2 (*df*)**	***p***	**n, % with symptom present**	**$N_{improved}$ / $N_{symptomatic}$ (%)**	**χ2 (*df*)**	***p***	**n, % with symptom present**	**$N_{improved}$ / $N_{symptomatic}$ (%)**	**χ2 (*df*)**	***p***	**n, % with symptom present**	**$N_{improved}$ / $N_{symptomatic}$ (%)**	**χ2 (*df*)**	***p***
Eating large amounts of food																
n = 4 5.0%	n = 4 14.8%	1/1 (100)	1.80 (1)	.18	n = 0 0%	0/0	[no cases]		n = 0 0%	0/0	[no cases]		n = 0 0%	0/0	[no cases]	
Loss of control eating																
n = 8 10%	n = 4 14.8%	0/2 (0)	2.00 (1)	.16	n = 4 18.2%	1/2 (50)	1.00 (1)	.32	n = 1 8.3%	0/1 (0)	[no discordant pairs]		n = 1 11.1%	1/1 (100)	0.00 (1)	1.00

**Baseline**	**Mid-intervention**				**Post**				**6-months follow-up**				**12-months follow-up**			
Excessive exercise																
n = 14<br>17.5%	n = 4<br>14.8%	3/5 (60)	0.20 (1)	.65	n = 2<br>9.1%	1/3 (33.3)	1.00 (1)	.32	n = 3<br>25.0%	1/4 (25)	1.00 (1)	.32	n = 1<br>11.1%	1/2 (50)	1.00 (1)	.32
Appetite suppressants																
n = 1<br>1.3%	n = 0<br>0%	1/1 (100)	[no cases]		n = 1<br>4.6%	1/1 (100)	0.00 (1)	1.00	n = 0<br>0%	0/0	[no cases]		n = 0<br>0%	1/1 (100)	[no cases]	
Fasting to maintain weight/figure																
n = 16<br>20.0%	n = 5<br>18.5%	2/6 (33.3)	0.33 (1)	.56	n = 5<br>22.7%	2/5 (40)	0.00 (1)	1.00	n = 4<br>33.3%	0/4 (0)	[no discordant pairs]		n = 4<br>44.4%	0/3 (0)	1.00 (1)	.32
Use of diuretics																
n = 0<br>0%	n = 0<br>0%	[no cases at baseline]	[no cases]		n = 0<br>0%	[no cases at baseline]	[no cases]		n = 0<br>0%	[no cases at baseline]	[no cases]		n = 0<br>0%	[no cases at baseline]	[no cases]	
Use of laxatives																
n = 0<br>0%	n = 0<br>0%	[no cases at baseline]	[no cases]		n = 1<br>4.6%	[no cases at baseline]	[no baseline cases for comparison]		n = 0<br>0%	[no cases at baseline]	[no cases]		n = 0<br>0%	[no cases at baseline]	[no cases]	
Induced vomiting																
n = 1<br>1.3%	n = 1<br>3.7%	0/0	1.00 (1)	.32	n = 0<br>0%	0/0	[no cases]		n = 1<br>8.3%	0/0	1.00 (1)	.32	n = 2<br>22.2%	0/0	2.00 (1)	.16
**Fruit and vegetable intake** (0-6)																
n = 80<br>**Median (Q1; Q3)**	n = 27<br>**Median (Q1; Q3)**	**Median difference, mean difference**	**S**	***p***	n = 22<br>**Median (Q1; Q3)**	**Median difference, mean difference**	**S**	***p***	n = 12<br>**Median (Q1; Q3)**	**Median difference, mean difference**	**S**	***p***	n = 9<br>**Median (Q1; Q3)**	**Median difference, mean difference**	**S**	***p***
Fresh fruit																
3 (1; 4)	3 (1; 4)	0 (median)<br>0.19 (mean)	15	.54	2 (1; 3)	0 (median)<br>0.41 (mean)	17	.13	2 (1; 2.5)	1 (median)<br>0.75 (mean)	14.5	.055	2 (1; 3)	1 (median)<br>0.89 (mean)	7	.19
Vegetables (fresh)																
3 (2; 4)	3 (2; 4)	0 (median)<br>-0.11 (mean)	-8	.46	3 (2; 4)	0 (median)<br>0 (mean)	-2	1.00	2 (1; 3.5)	0 (median)<br>-0.25 (mean)	-4.5	.63	2 (2; 3)	-1 (median)<br>-0.78 (mean)	-11	.09
Vegetables (frozen)																
0 (0; 1)	0 (0; 1)	0 (median)<br>-0.15 (mean)	-5.5	.48	0 (0; 1)	0 (median)<br>-0.14 (mean)	-2	.86	0 (0; 1)	0 (median)<br>0 (mean)	0	1.00	1 (0; 2)	0 (median)<br>0.56 (mean)	3	.66
Smoothies/juice																
0.5 (0; 1)	1 (0; 3)	0 (median)<br>0.15 (mean)	10.5	.45	1 (1; 2)	0 (median)<br>0.23 (mean)	10	.45	1 (0; 1.5)	0 (median)<br>-0.08 (mean)	-1	.75	1 (1; 3)	0 (median)<br>0.33 (mean)	2	.63

Note. All p-values represent comparisons with baseline.

Primary and secondary outcomes study arm Fit (completer sample)

Table S10

Secondary outcomes in study arm Fit (completer sample)

**Baseline**	**Mid-intervention**				**Post**				**6-months follow-up**				**12-months follow-up**			
**n, mean (SD)**	**Mean (SD)**	**Cohen's *d* (95%CI)**	***t* statistic (*df*)**	**p**	**Mean (SD)**	**Cohen's *d* (95%CI)**	***t* statistic (*df*)**	**p**	**Mean (SD)**	**Cohen's *d* (95%CI)**	***t* statistic (*df*)**	**p**	**Mean (SD)**	**Cohen's *d* (95%CI)**	***t* statistic (*df*)**	**p**
Weight Concerns (WCS, 0-100)																
n = 1339<br>47.43<br>(16.54)	n = 453<br>43.37<br>(14.66)	-0.28<br>(-0.37, -0.18)	-5.90<br>(452)	<.001	n = 294<br>38.71<br>(15.16)	-0.54<br>(-0.67, -0.42)	-9.32<br>(293)	<.001	n = 160<br>36.75<br>(15.65)	-0.70<br>(-0.88, -0.53)	-8.90 (159)	<.001	n = 111<br>35.75<br>(15.22)	-0.68<br>(-0.89, -0.47)	-7.20<br>(110)	<.001
Eating Disorder Examination Questionnaire total score (EDE-Q, 0-6)																
n = 1339<br>2.29<br>(0.99)	n = 453<br>1.82<br>(0.90)	-0.52<br>(-0.61, -0.42)	-10.98<br>(452)	<.001	n = 294<br>1.50<br>(0.92)	-0.77<br>(-0.90, -0.64)	-13.29<br>(293)	<.001	n = 158<br>1.39<br>(0.88)	-0.89<br>(-1.08, -0.71)	-11.24<br>(157)	<.001	n = 111<br>1.32<br>(0.96)	-0.87<br>(-1.09, -0.65)	-9.16<br>(110)	<.001
Intuitive Eating Scale (IES, 1-5)																
n = 1339<br>3.08<br>(0.53)	n = 448<br>3.33<br>(0.55)	0.57 (0.47, 0.67)	12.04<br>(447)	<.001	n = 273<br>3.47<br>(0.60)	0.71 (0.58, 0.84)	11.73<br>(272)	<.001	n = 143<br>3.55<br>(0.62)	0.84 (0.64, 1.03)	10.00<br>(142)	<.001	n = 100<br>3.60<br>(0.62)	0.90 (0.66, 1.13)	8.97 (99)	<.001
Rosenberg Self-Esteem Scale (RSE, 10-40)																
n = 1339<br>31.62<br>(5.80)	n = 449<br>32.65<br>(5.67)	0.20 (0.11, 0.30)	4.29	<.001	n = 274<br>33.58<br>(5.18)	0.45 (0.32, 0.57)	7.37<br>(273)	<.001	n = 145<br>34.14<br>(5.15)	0.41 (0.24, 0.58)	4.95 (144)	<.001	n = 102<br>35.07<br>(4.49)	0.54 (0.33, 0.75)	5.44<br>(101)	<.001
Assessment of Quality of Life-8D (AQoL-8D, 1-5)																
n = 1130<br>0.69<br>(0.16)	[not assessed]				n = 272<br>0.75<br>(0.17)	0.40 (0.27, 0.52)	6.51<br>(266)	<.001	n = 144<br>0.76<br>(0.16)	0.53 (0.35, 0.70)	6.27 (141)	<.001	n = 102<br>0.78<br>(0.15)	0.52 (0.32, 0.73)	5.27<br>(100)	<.001
BMI																
n = 1338<br>30.51<br>(5.02)	[not assessed]				n = 291<br>29.96<br>(4.94)	-0.13<br>(-0.25, -0.02)	-2.23<br>(290)	.027	n = 155<br>29.93<br>(5.24)	-0.16<br>(-0.31, 0.002)	-1.95 (154)	.054	n = 107<br>29.44<br>(4.26)	-0.20<br>(-0.39, -0.01)	-2.07<br>(106)	.041
**Presence of ED Core symptoms (yes vs. no), past 4 weeks**																
n = 1339	n = 452				n = 292				n = 155				n = 109			
**n, % with symptom present**	**n, % with symptom present**	**$N_{improved}$ / $N_{symptomatic}$ (%)**	**χ2 (*df*)**	***p***	**n, % with symptom present**	**$N_{improved}$ / $N_{symptomatic}$ (%)**	**χ2 (*df*)**	***p***	**n, % with symptom present**	**$N_{improved}$ / $N_{symptomatic}$ (%)**	**χ2 (*df*)**	***p***	**n, % with symptom present**	**$N_{improved}$ / $N_{symptomatic}$ (%)**	**χ2 (*df*)**	***p***
Eating large amounts of food																
n = 69<br>5.2%	n = 35<br>7.7%	13/18 (72.2)	6.72 (1)	.010	n = 24<br>8.2%	6/10 (60)	7.54 (1)	.006	n = 11<br>7.1%	4/5 (80)	2.57 (1)	.11	n = 7<br>6.4%	2/2 (100)	2.78 (1)	.10
Loss of control eating																
n = 201<br>15.0%	n = 66<br>14.6%	43/69 (62.3)	0.12 (1)	.74	n = 31<br>10.6%	31/42 (73.8)	2.37 (1)	.12	n = 21<br>13.6%	13/17 (76.5)	0.53 (1)	.47	n = 14<br>12.8%	7/12 (58.3)	0.25 (1)	.62

Baseline	Mid-intervention				Post				6-months follow-up				12-months follow-up			
Excessive exercise																
n = 31<br>2.3%	n = 7<br>1.6%	11/14 (78.6)	3.27 (1)	.07	n = 4<br>1.4%	7/8 (87.5)	1.60 (1)	.21	n = 3<br>1.9%	5/6 (83.3)	1.29 (1)	.26	n =2<br>1.8%	6/6 (100)	2.00 (1)	.16
Fasting to maintain weight/figure																
n = 185<br>13.8%	n = 28<br>6.2%	43/59 (72.9)	17.47 (1)	<.001	n = 25<br>8.6%	23/36 (63.9)	3.46 (1)	.06	n = 15<br>9.7%	22/23 (95.7)	1.78 (1)	.18	n = 13<br>11.9%	14/18 (77.8)	1.09 (1)	.30
Appetite suppressants																
n = 11<br>0.8%	n = 1<br>0.2%	7/7 (100)	4.50 (1)	.034	n = 1<br>0.3%	2/2 (100)	0.33 (1)	.56	n = 0<br>0%	3/3 (100)	[No cases]		n = 0<br>0%	1/1 (100)	[No cases]	
Use of diuretics																
n = 6<br>0.5%	n = 3<br>0.7%	4/5 (80)	0.67 (1)	.41	n = 0<br>0.0%	2/2 (100)	[No cases]		n = 1<br>0.7%	0/0	1.00 (1)	.32	n = 0<br>0.0%	0/0	[No cases]	
Use of laxatives																
n = 4<br>0.3%	n = 0<br>0.0%	1/1 (100)	[No cases]		n = 0<br>0.0%	1/1 (100)	[No cases]		n = 0<br>0.0%	0/0	[No cases]		n = 0<br>0.0%	0/0	[No cases]	
Induced vomiting																
n = 5<br>0.4%	n = 0<br>0.0%	2/2 (100)	[No cases]		n = 0<br>0.0%	2/2 (100)	[No cases]		n = 1<br>0.7%	0/1 (0)	[no discordant pairs]		n = 0<br>0.0%	0/0	[No cases]	
**Fruit and vegetable intake** (0-6)																
n = 1339	n = 450				n = 271				n = 144				n = 101			
**Median (Q1; Q3)**	**Median (Q1; Q3)**	**Median difference, mean difference**	**S**	***p***	**Median (Q1; Q3)**	**Median difference, mean difference**	**S**	***p***	**Median (Q1; Q3)**	**Median difference, mean difference**	**S**	***p***	**Median (Q1; Q3)**	**Median difference, mean difference**	**S**	***p***
Fresh fruit																
3 (1; 3)	4 (3; 4)	1 (median)<br>0.80 (mean)	16199.5	<.001	4 (2; 4)	1 (median)<br>0.80 (mean)	5916	<.001	3 (2; 4)	1 (median)<br>0.72 (mean)	1612.5	<.001	3 (2; 4)	0 (median)<br>0.46 (mean)	577	<.001
Vegetables (fresh)																
3 (1; 3)	3 (2; 4)	0 (median)<br>0.55 (mean)	11315.5	<.001	3 (2; 4)	1 (median)<br>0.65 (mean)	5716.5	<.001	3 (2; 4)	1 (median)<br>0.60 (mean)	1370	<.001	3 (2; 4)	1 (median)<br>0.60 (mean)	766.5	<.001
Vegetables (frozen)																
1 (0; 1)	1 (0; 1)	0 (median)<br>0.19 (mean)	3136	<.001	1 (0; 1)	0 (median)<br>0.14 (mean)	882	.013	1 (0; 1)	0 (median)<br>0.06 (mean)	114.5	.46	1 (0; 1)	0 (median)<br>0.19 (mean)	170	.06
Smoothies/juice																
1 (0; 1)	1 (0; 2)	0 (median)<br>0.14 (mean)	1836.5	.029	1 (0; 2)	0 (median)<br>0.14 (mean)	590	.11	1 (0; 1)	0 (median)<br>0.14 (mean)	221.5	.13	1 (0; 1)	0 (median)<br>-0.04 (mean)	-11.5	.90

Note. All p-values represent comparisons with baseline.

Secondary outcomes (figures)

Figure S2

Box plots of Eating Disorder Examination-Questionnaire scores for each time point and study arm

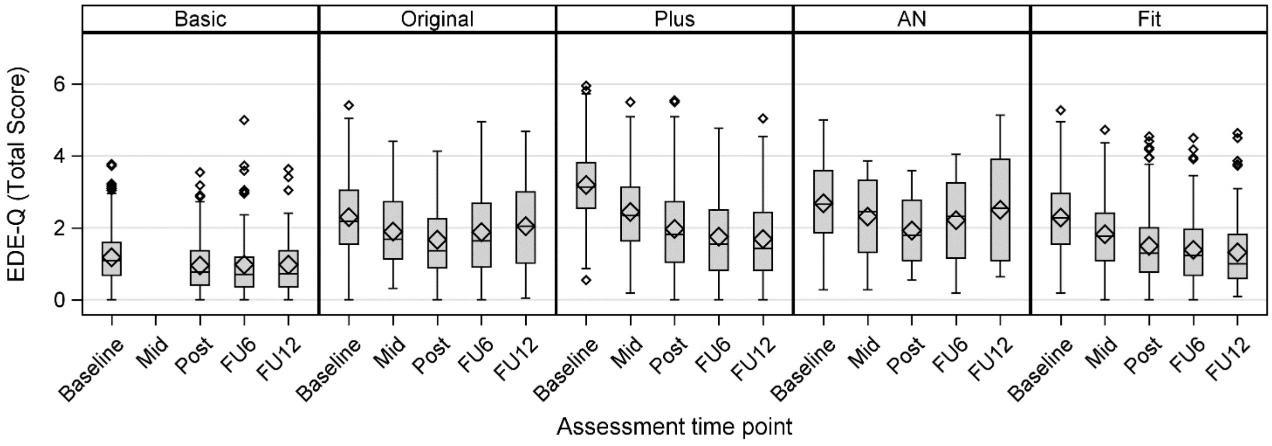

Figure S3

Box plots of Intuitive Eating Scale scores for each time point and study arm

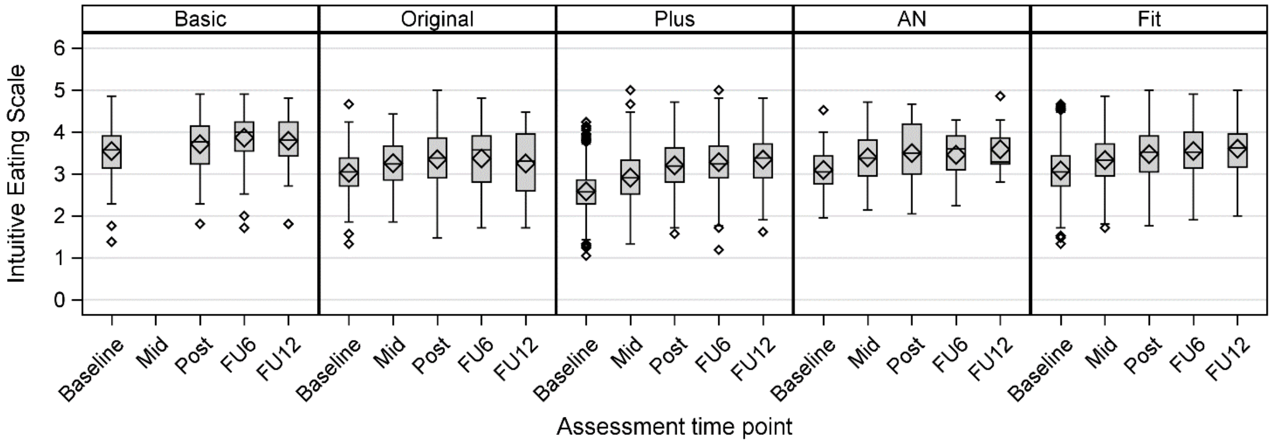

Figure S4

Box plots of Rosenberg Self-Esteem Scale scores for each time point and study arm

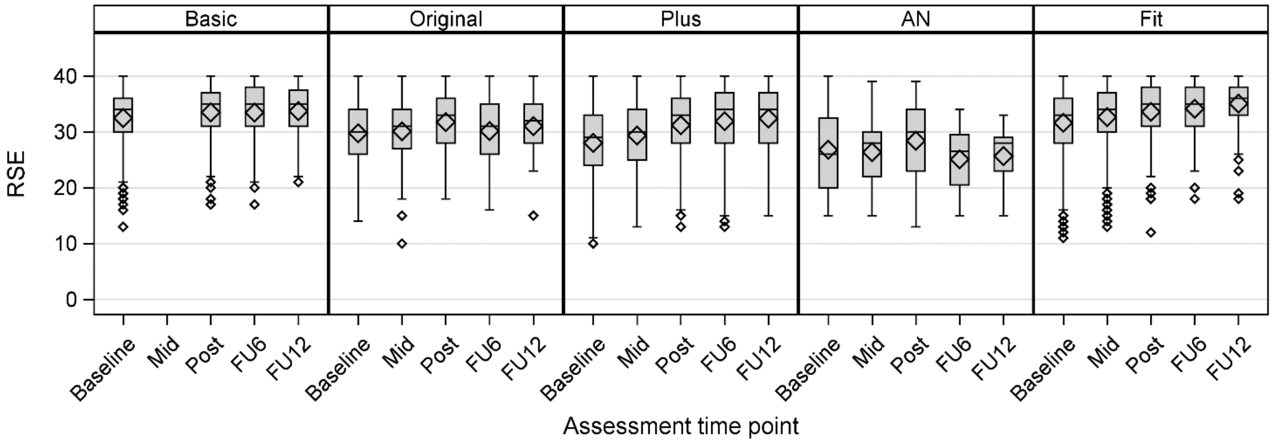

Figure S5

Box plots of Assessment of Quality of Life-8D scores for each time point and study arm

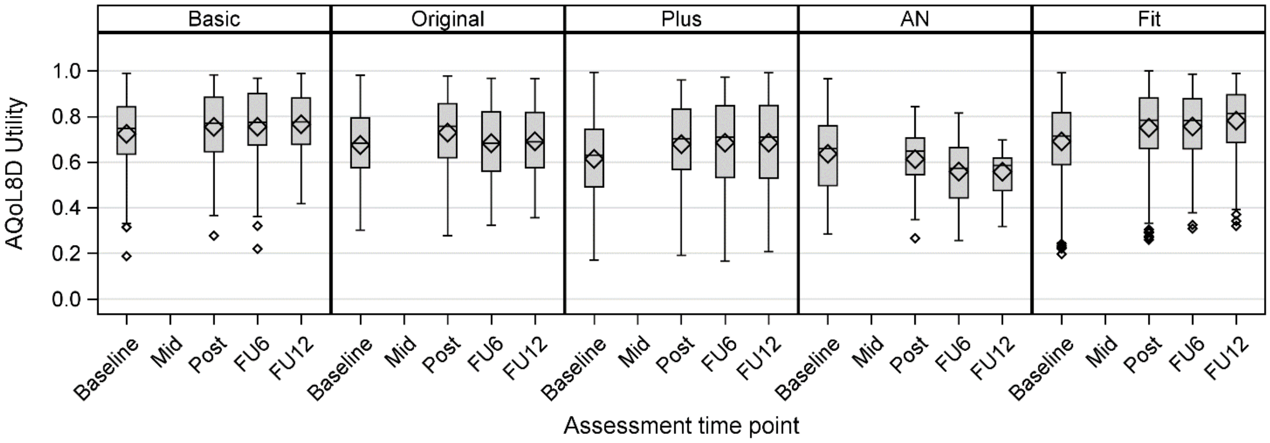

Sensitivity analyses using multiple imputed datasets

Table S11

Study arm Basic: Pooled (Rubin's rule) within-subjects effect sizes from baseline to post, and follow up assessments and results of t-tests for the primary outcome based on the ITT analysis in the multiple imputed dataset (sensitivity analyses)

	Mean Mid-baseline difference (SE)	*t* statistic	*p*	Cohen's *d* (95% CI)	Mean post-baseline differences (SE)	*t* statistic	*p*	Cohen's *d* (95% CI)	Mean FU6-baseline differences (SE)	*t* statistic	*p*	Cohen's *d* (95% CI)	Mean FU12-baseline differences (SE)	*t* statistic	*p*	Cohen's *d* (95% CI)
WCS	[not assessed]				-1.49 (0.56)	-2.66	.008	-0.16 (-0.23, -0.08)	-1.30 (0.95)	-1.37	.17	-0.09 (-0.19, 0.01)	-0.02 (1.00)	-0.02	0.99	-0.00 (-0.10, 0.10)
EDE-Q	[not assessed]				-0.22 (0.03)	-6.42	<.001	-0.37 (-0.43, -0.30)	-0.22 (0-06)	-3.65	<.001	-0.24 (-0.35, -0.14)	-0.29 (0.04)	-7.14	<.001	-0.41 (-0.49, -0.32)
IES	[not assessed]				0.18 (0.02)	7.58	<.001	0.43 (0.37, 0.50)	0.32 (0.03)	11.92	<.001	0.77 (0.65, 0.90)	0.34 (0.03)	11.81	<.001	0.75 (0.62, 0.87)
RSE	[not assessed]				0.64 (0.21)	3.01	.003	0.18 (0.10, 0.26)	0.89 (0.23)	3.89	<.001	0.24 (0.15, 0.32)	1.03 (0.32)	3.23	.002	0.24 (0.12, 0.37)
AQoL-8D	[not assessed]				0.03 (0.01)	4.94	<.001	0.29 (0.22, 0.37)	0.04 (0.01)	4.29	<.001	0.32 (0.19, 0.45)	0.05 (0.01)	5.47	<.001	0.41 (0.27, 0.55)

Note. Number of Imputations = 50; Imputation method: Fully Conditional Specification (FCS) and Predictive Mean Matching (PMM); Imputation model included WCS total score, age, BMI, EDEQ total score, PHQ-9 score, GAD-7 score.

Negative effect sizes indicate improvement in weight and shape concerns.

Abbreviations: WCS, Weight and Shape Concerns Scale; EDE-Q, Eating Disorder Examination-Questionnaire; IES, Intuitive Eating Scale; RSE, Rosenberg Self-Esteem Scale; AQoL-8D, Assessment of Quality of Life-8D

Table S12

Study arm Original: Pooled (Rubin's rule) within-subjects effect sizes from baseline to mid, post, and follow up assessments and results of t-tests for the primary outcome based on the ITT analysis in the multiple imputed dataset (sensitivity analyses)

	Mean Mid-baseline difference (SE)	*t* statistic	*p*	Cohen's *d* (95% CI)	Mean post-baseline differences (SE)	*t* statistic	*p*	Cohen's *d* (95% CI)	Mean FU6-baseline differences (SE)	*t* statistic	*p*	Cohen's *d* (95% CI)	Mean FU12-baseline differences (SE)	*t* statistic	*p*	Cohen's *d* (95% CI)
WCS	-2.44 (0.88)	-2.79	.006	-0.19 (-0.29, -0.09)	-7.22 (0.95)	-7.63	<.001	-0.50 (-0.60, -0.41)	-4.01 (1.32)	-3.04	.003	-0.22 (-0.33, -0.11)	-5.06 (1.65)	-3.07	.003	-0.24 (-0.37, -0,11)
EDE-Q	-0.39 (0.05)	-8.09	<.001	-0.49 (-0.57, -0.40)	-0.60 (0.06)	-10.21	<.001	-0.67 (-0,79, -0,55)	-0.44 (0.09)	-4.87	<.001	-0.37 (-0.51, -0.24)	-0.33 (0.14)	-2.44	.017	-0.24 (-0.43, -0.05)
IES	0.20 (0.03)	7.01	<.001	0.44 (0.35, 0.52)	0.29 (0.03)	8.63	<.001	0.57 (0.46, 0.68)	0.32 (0.06)	5.47	<.001	0.47 (0.30, 0.65)	0.21 (0.07)	3.13	.002	0.27 (0.12, 0.43)
RSE	0.48 (0.26)	1.88	.06	0.12 (0.04, 0.19)	1.86 (0.26)	7.18	<.001	0.44 (0.36, 0.51)	0.46 (0.42)	1.07	.29	0.10 (-0.06, 0.25)	1.44 (0.51)	2.85	.005	0.24 (0.11, 0.38)
AQoL-8D	[not assessed]				0.04 (0.01)	5.22	<.001	0.38 (0.28, 0.49)	0.02 (0.01)	1.88	.06	0.16 (-0.02, 0.35)	0.01 (0.01)	1.22	.23	0.09 (-0.02, 0.21)

Note. Number of Imputations = 50; Imputation method: Fully Conditional Specification (FCS) and Predictive Mean Matching (PMM); Imputation model included WCS total score, age, BMI, EDEQ total score, PHQ-9 score, GAD-7 score.

Negative effect sizes indicate improvement in weight and shape concerns.

Abbreviations: WCS, Weight and Shape Concerns Scale; EDE-Q, Eating Disorder Examination-Questionnaire; IES, Intuitive Eating Scale; RSE, Rosenberg Self-Esteem Scale; AQoL-8D, Assessment of Quality of Life-8D

Table S13

Study arm Plus: Pooled (Rubin's rule) within-subjects effect sizes from baseline to mid, post, and follow up assessments and results of t-tests for the primary outcome based on the ITT analysis in the multiple imputed dataset (sensitivity analyses)

	Mean Mid-baseline difference (SE)	*t* statistic	*p*	Cohen's *d* (95% CI)	Mean post-baseline differences (SE)	*t* statistic	*p*	Cohen's *d* (95% CI)	Mean FU6-baseline differences (SE)	*t* statistic	*p*	Cohen's *d* (95% CI)	Mean FU12-baseline differences (SE)	*t* statistic	*p*	Cohen's *d* (95% CI)
WCS	-6.08 (0.42)	-14.58	<.001	-0.50 (-0.55, -0.46)	-12.59 (0.56)	-22.61	<.001	-0.84 (-0.92, -0.77)	-15.40 (0.80)	-19.36	<.001	-0.84 (-0.94, -0.74)	-16.94 (1.00)	-16.87	<.001	-0.94 (-1.07, -0.81)
EDE-Q	-0.80 (0.03)	-23.68	<.001	-0.94 (-1.00, -0.88)	-1.24 (0.04)	-29.61	<.001	-1.14 (-1.21, -1.06)	-1.26 (0.05)	-23.26	<.001	-1.02 (-1.13, -0.92)	-1.33 (0.07)	-18.29	<.001	-1.10 (-1.28, -0.93)
IES	0.37 (0.02)	20.68	<.001	0.76 (0.70, 0.82)	0.63 (0.02)	26.32	<.001	0.99 (0.93, 1.07)	0.60 (0.03)	18.49	<.001	0.90 (0.79, 1.00)	0.65 (0.04)	15.71	<.001	1.00 (0.82, 1.19)
RSE	1.35 (0.14)	9.67	<.001	0.34 (0.29, 0.39)	2.75 (0.16)	16.66	<.001	0.61 (0.56, 0.66)	3.41 (0.2)	15.80	<.001	0.68 (0.59, 0.78)	3.62 (0.27)	13.43	<.001	0.70 (0.57, 0.89)
AQoL-8D	[not assessed]				0.06 (0.004)	15.17	<.001	0.56 (0.50, 0.63)	0.07 (0.01)	11.98	<.001	0.58 (0.46, 0.69)	0.07 (0.01)	9.71	<.001	0.52 (0.40, 0.63)

Note. Number of Imputations = 50; Imputation method: Fully Conditional Specification (FCS) and Predictive Mean Matching (PMM); Imputation model included WCS total score, age, BMI, EDEQ total score, PHQ-9 score, GAD-7 score.

Negative effect sizes indicate improvement in weight and shape concerns.

Abbreviations: WCS, Weight and Shape Concerns Scale; EDE-Q, Eating Disorder Examination-Questionnaire; IES, Intuitive Eating Scale; RSE, Rosenberg Self-Esteem Scale; AQoL-8D, Assessment of Quality of Life-8D

Table S14

Study arm AN: Pooled (Rubin's rule) within-subjects effect sizes from baseline to mid, post, and follow up assessments and results of t-tests for the primary outcome based on the ITT analysis in the multiple imputed dataset (sensitivity analyses)

	Mean Mid-baseline difference (SE)	*t* statistic	*p*	Cohen's *d* (95% CI)	Mean post-baseline differences (SE)	*t* statistic	*p*	Cohen's *d* (95% CI)	Mean FU6-baseline differences (SE)	*t* statistic	*p*	Cohen's *d* (95% CI)	Mean FU12-baseline differences (SE)	*t* statistic	*p*	Cohen's *d* (95% CI)
WCS	-6.65 (4.05)	-1.64	.10	-0.38 (-0.79, 0.03)	-12.03 (3.31)	-3.64	<.001	-0.75 (-1.09, -0.41)	-11.06 (6.30)	-1.76	.08	-0.38 (-0.75, -0.00)	-8.12 (4.40)	-1.85	.07	-0.35 (-0.69, -0.01)
EDE-Q	-0.71 (0.25)	-2.82	.006	-0.64 (-1.07, -0.20)	-0.83 (0.23)	-3.70	<.001	-0.69 (-1.04, -0.34)	-0.81 (0.38)	-2.16	.033	-0.49 (-0.92, -0.07)	-0.49 (0.28)	-1.78	.08	-0.33 (-0.63, -0.03)
IES	0.31 (0.14)	2.24	.028	0.51 (0.11, 0.91)	0.31 (0.16)	1.90	.06	0.35 (0.06, 0.63)	0.50 (0.15)	3.33	.001	0.58 (0.29, 0.87)	0.51 (0.10)	5.38	<.001	1.12 (0.71, 1.53)
RSE	0.88 (1.03)	0.86	.39	0.17 (-0.16, 0.50)	2.49 (1.46)	1.71	.09	0.29 (0.02, 0.57)	0.10 (1.62)	0.06	.95	0.01 (-0.27, 0.29)	-0.25 (1.02)	-0.25	.81	-0.04 (-0.23, 0.16)
AQoL-8D	[not assessed]				0.02 (0.03)	0.56	.58	0.12 (-0.19, 0.42)	-0.05 (0.04)	-1.14	.26	-0.18 (-0.40, 0.04)	-0.05 (0.02)	-2.32	.02	-0.32 (-0.47, -0.16)

Note. Number of Imputations = 50; Imputation method: Markov chain Monte Carlo (MCMC); Imputation model included WCS total score, age, BMI, EDEQ total score, PHQ-9 score, GAD-7 score.

Negative effect sizes indicate improvement in weight and shape concerns.

Abbreviations: WCS, Weight and Shape Concerns Scale; EDE-Q, Eating Disorder Examination-Questionnaire; IES, Intuitive Eating Scale; RSE, Rosenberg Self-Esteem Scale; AQoL-8D, Assessment of Quality of Life-8D

Table S15

Study arm Fit: Pooled (Rubin's rule) within-subjects effect sizes from baseline to mid, post, and follow up assessments and results of t-tests for the primary outcome based on the ITT analysis in the multiple imputed dataset (sensitivity analyses)

	Mean Mid-baseline difference (SE)	*t* statistic	*p*	Cohen's *d* (95% CI)	Mean post-baseline differences (SE)	*t* statistic	*p*	Cohen's *d* (95% CI)	Mean FU6-baseline differences (SE)	*t* statistic	*p*	Cohen's *d* (95% CI)	Mean FU12-baseline differences (SE)	*t* statistic	*p*	Cohen's *d* (95% CI)
WCS	-3.27 (0.38)	-8.60	<.001	-0.30 (-0.34, -0.26)	-7.80 (0.50)	-15.75	<.001	-0.56 (-0.62, -0.51)	-8.54 (0.70)	-12.12	<.001	-0.55 (-0.64, -0.45)	-9.32 (0.79)	-11.79	<.001	-0.56 (-0.66, -0.46)
EDE-Q	-0.42 (0.03)	-15.34	<.001	-0.53 (-0.58, -0.48)	-0.74 (0.04)	-20.44	<.001	-0.80 (-0.87, -0.73)	-0.75 (0.05)	-16.42	<.001	-0.77 (-0.87, -0.66)	-0.76 (0.06)	-13.38	<.001	-0.73 (-0.87, -0.59)
IES	0.24 (0.02)	14.37	<.001	0.55 (0.49, 0.60)	0.39 (0.02)	16.62	<.001	0.71 (0.65, 0.77)	0.41 (0.03)	16.39	<.001	0.76 (0.67, 0.85)	0.44 (0.04)	11.70	<.001	0.74 (0.61, 0.88)
RSE	0.72 (0.12)	5.78	<.001	0.21 (0.17, 0.25)	1.73 (0.14)	12.19	<.001	0.47 (0.41, 0.53)	1.91 (0.19)	9.98	<.001	0.43 (0.35, 0.50)	2.45 (0.24)	10.23	<.001	0.52 (0.41, 0.63)
AQoL-8D	[not assessed]				0.04 (0.00)	10.68	<.001	0.41 (0.36, 0.47)	0.05 (0.01)	9.56	<.001	0.47 (0.38, 0.56)	0.06 (0.01)	8.56	<.001	0.52 (0.39, 0.64)

Note. Number of Imputations = 50; Imputation method: Fully Conditional Specification (FCS) and Predictive Mean Matching (PMM); Imputation model included WCS total score, age, BMI, EDEQ total score, PHQ-9 score, GAD-7 score.

Negative effect sizes indicate improvement in weight and shape concerns.

Abbreviations: WCS, Weight and Shape Concerns Scale; EDE-Q, Eating Disorder Examination-Questionnaire; IES, Intuitive Eating Scale; RSE, Rosenberg Self-Esteem Scale; AQoL-8D, Assessment of Quality of Life-8D

Sensitivity analyses using mixed model analyses

Table S16

Results of mixed model analyses with comparisons from baseline to mid, post, and follow up assessments based on completer data

	Baseline	Mid	Baseline-mid comparison		Post	Baseline-post comparison		6-months follow-up	Baseline-FU6 comparison		12-months follow-up	Baseline-FU12 comparison	
	Estimated mean (SE)	Estimated mean (SE)	*t* statistic	*p*	Estimated mean (SE)	*t* statistic	*p*	Estimated mean (SE)	*t* statistic	*p*	Estimated mean (SE)	*t* statistic	*p*
**Study arm Basic**													
WCS	29.09 (0.56)	[not assessed]			27.82 (0.75)	-1.17	.97	27.84 (1.05)	0.14	1.00	29.28 (1.29)	-52.30	<.001
EDE-Q	1.18 (0.03)	[not assessed]			0.96 (0.05)	-3.02	.011	0.98 (0.06)	-3.27	.005	0.91 (0.08)	-35.15	<.001
IES	3.55 (0.03)	[not assessed]			3.72 (0.03)	6.58	<.001	3.83 (0.05)	5.69	<.001	3.87 (0.06)	-135.11	<.001
RSE	32.49 (0.25)	[not assessed]			32.94 (0.33)	1.95	.21	33.32 (0.44)	2.38	.07	33.82 (0.55)	-128.89	<.001
AQoL-8D	0.72 (0.01)	[not assessed]			0.75 (0.01)	4.12	<.001	0.77 (0.01)	4.08	<.001	0.77 (0.01)	3.30	.003
**Study arm Original**													
WCS	51.12 (0.81)	49.61 (1.15)	-1.52	.52	44.63 (1.34)	-4.98	<.001	47.72 (1.87)	-1.79	.30	45.63 (2.23)	-2.41	.07
EDE-Q	2.30 (0.05)	1.96 (0.08)	-5.07	<.001	1.74 (0.09)	-6.42	<.001	1.87 (0.12)	-3.39	.003	2.01 (0.15)	-1.92	.23
IES	3.03 (0.03)	3.20 (0.04)	4.51	<.001	3.31 (0.05)	5.56	<.001	3.28 (0.07)	3.35	.004	3.21 (0.09)	2.04	.17
RSE	29.73 (0.30)	29.99 (0.40)	0.78	1.00	31.42 (0.48)	3.80	<.001	30.14 (0.65)	0.62	1.00	31.09 (0.79)	1.69	.37
AQoL-8D	0.67 (0.01)	[not assessed]			0.72 (0.01)	3.58	.001	0.70 (0.02)	1.24	.65	0.70 (0.02)	0.99	.97
**Study arm Plus**													
WCS	61.90 (0.38)	56.35 (0.59)	-10.18	<.001	49.31 (0.71)	-17.46	<.001	46.54 (0.99)	-15.17	<.001	44.07 (1.24)	-14.00	<.001
EDE-Q	3.19 (0.03)	2.42 (0.04)	-21.57	<.001	2.00 (0.05)	-24.81	<.001	1.95 (0.07)	-18.29	<.001	1.83 (0.08)	-15.94	<.001
IES	2.59 (0.01)	2.92 (0.02)	15.59	<.001	3.18 (0.03)	20.99	<.001	3.18 (0.04)	14.89	<.001	3.26 (0.05)	13.60	<.001
RSE	28.04 (0.17)	29.17 (0.22)	6.59	<.001	30.54 (0.27)	10.18	<.001	31.30 (0.37)	9.17	<.001	31.48 (0.47)	7.43	<.001
AQoL-8D	0.61 (0.00)	[not assessed]			0.67 (0.01)	9.61	<.001	0.69 (0.01)	8.35	<.001	0.68 (0.01)	6.20	<.001
**Study arm AN**													
WCS	58.85 (1.60)	54.69 (2.25)	-2.05	.18	48.14 (2.65)	-3.99	<.001	49.32 (3.46)	-2.65	.04	52.63 (4.19)	-1.43	.63
EDE-Q	2.68 (0.13)	2.17 (0.17)	-3.74	.002	1.78 (0.20)	-4.81	<.001	2.17 (0.25)	-2.01	.20	2.43 (0.31)	-0.79	1.00
IES	3.10 (0.06)	3.32 (0.09)	3.01	.015	3.49 (0.10)	3.90	<.001	3.44 (0.13)	2.59	.048	3.41 (0.16)	1.93	.23
RSE	26.80 (0.78)	27.46 (1.00)	0.84	1.00	29.03 (1.17)	2.04	.18	27.48 (1.50)	0.46	1.00	26.44 (1.82)	-0.19	1.00
AQoL-8D	0.64 (0.02)	[not assessed]			0.64 (0.03)	-0.05	1.00	0.59 (0.04)	-0.99	.98	0.57 (0.05)	-1.25	.66
**Study arm Fit**													
WCS	47.43 (0.45)	44.34 (0.60)	-6.19	<.001	39.42 (0.75)	-10.94	<.001	38.77 (1.00)	-8.61	<.001	38.67 (1.25)	-6.86	<.001
EDE-Q	2.29 (0.03)	1.88 (0.04)	-12.70	<.001	1.57 (0.05)	-15.58	<.001	1.54 (0.06)	-11.80	<.001	1.51 (0.08)	-9.80	<.001

	Baseline	Mid	Baseline-mid comparison		Post	Baseline-post comparison		6-months follow-up	Baseline-FU6 comparison		12-months follow-up	Baseline-FU12 comparison	
	**Estimated mean (SE)**	**Estimated mean (SE)**	***t* statistic**	***p***	**Estimated mean (SE)**	***t* statistic**	***p***	**Estimated mean (SE)**	***t* statistic**	***p***	**Estimated mean (SE)**	***t* statistic**	***p***
IES	3.08 (0.01)	3.33 (0.02)	13.64	<.001	3.44 (0.03)	13.49	<.001	3.48 (0.04)	10.62	<.001	3.52 (0.05)	9.28	<.001
RSE	31.62 (0.16)	32.32 (0.19)	4.96	<.001	33.09 (0.24)	6.83	<.001	33.44 (0.32)	5.97	<.001	33.67 (0.40)	5.20	<.001
AQoL-8D	0.69 (0.00)	[not assessed]			0.73 (0.01)	7.20	<.001	0.74 (0.01)	6.03	<.001	0.75 (0.01)	5.52	<.001

Note. *p* is Bonferroni adjusted for multiple testing. WCS scores, negative effect sizes indicate improvement in weight and shape concerns.

Abbreviations: WCS, Weight and Shape Concerns Scale; EDE-Q, Eating Disorder Examination-Questionnaire; IES, Intuitive Eating Scale; RSE, Rosenberg Self-Esteem Scale; AQoL-8D, Assessment of Quality of Life-8D

Relationship of adherence and outcomes

Table S17

Study arm Basic: Results of simple linear regression to predict WCS at post, 6-months follow-up, and at 12-months follow-up based on number of completed session

Variable	df	Parameter estimate (SE)	95% CI	*t* statistic	*p*
**WCS Baseline – Post, n = 178**					
Intercept	1	4.15 (5.71)	-7.12, 15.42	0.73	.47
Completed sessions	1	-1.40 (1.47)	-4.30, 1.49	-0.96	.34
**WCS Baseline – 6-months follow-up, n = 88**					
Intercept	1	40.12 (10.40)	19.46, 60.79	3.86	<.001
Completed sessions	1	-10.54 (2.63)	-15.78, -5.30	-4.00	<.001
**WCS Baseline – 12-months follow-up, n = 65**					
Intercept	1	-3.33 (15.24)	-33.79, 27.13	-0.22	.83
Completed sessions	1	0.82 (3.84)	-6.85, 8.49	0.21	.83

Note. Abbreviations: WCS, Weight and Shape Concerns Scale

Table S18

Study arm Original: Results of simple linear regression to predict WCS at post, 6-months follow-up, and at 12-months follow-up based on number of completed session

Variable	df	Parameter estimate (SE)	95% CI	*t* statistic	*p*
**WCS Baseline – Post, n = 97**					
Intercept	1	8.40 (5.79)	-3.09, 19.89	1.45	.15
Completed sessions	1	-2.01 (0.77)	-3.55, -0.48	-2.61	.011
**WCS Baseline – 6-months follow-up, n = 41**					
Intercept	1	0.87 (17.05)	-33.62, 35.36	0.05	.96
Completed sessions	1	-0.59 (2.20)	-5.04, 3.86	-0.27	.79
**WCS Baseline – 12-months follow-up, n = 36**					
Intercept	1	7.79 (8.61)	-9.72, 25.29	0.90	.37
Completed sessions	1	-2.04 (1.17)	-4.42, 0.34	-1.74	.09

Note. Abbreviations: WCS, Weight and Shape Concerns Scale

Table S19

Study arm Plus: Results of simple linear regression to predict WCS at post, 6-months follow-up, and at 12-months follow-up based on number of completed session

Variable	df	Parameter estimate (SE)	95% CI	*t* statistic	*p*
**WCS Baseline – Post, n = 301**					
Intercept	1	-1.96 (3.01)	-7.88, 3.97	-0.65	.52
Completed sessions	1	-1.59 (0.42)	-2.41, -0.77	-3.80	<.001
**WCS Baseline – 6-months follow-up, n = 145**					
Intercept	1	-7.32 (6.22)	-19.62, 4.98	-1.18	.24
Completed sessions	1	-1.39 (0.83)	-3.03, 0.24	-1.68	.09
**WCS Baseline – 12-months follow-up, n = 100**					
Intercept	1	-2.58 (9.72)	-21.86, 16.70	-0.27	.79
Completed sessions	1	-2.23 (1.27)	-4.74, 0.29	0.08	.08

Note. Abbreviations: WCS, Weight and Shape Concerns Scale

Table S20

Study arm AN: Results of simple linear regression to predict WCS at post, 6-months follow-up, and at 12-months follow-up based on number of completed session

Variable	df	Parameter estimate (SE)	95% CI	*t* statistic	*p*
**WCS Baseline – Post, n = 22**					
Intercept	1	6.48 (5.40)	-4.80, 17.75	1.20	.24
Completed sessions	1	-2.04 (0.63)	-3.35, -0.72	-3.23	.004
**WCS Baseline – 6-months follow-up, n = 12**					
Intercept	1	-8.73 (38.37)	-94.22, 76.77	-0.23	.82
Completed sessions	1	-0.44 (4.01)	-9.37, 8.49	-0.11	.91
**WCS Baseline – 12-months follow-up, n = 9**					
Intercept	1	-8.52 (6.62)	-23.78, 6.74	-1.29	.23
Completed sessions	1	[not estimable]			

Note. Abbreviations: WCS, Weight and Shape Concerns Scale

Table S21

Study arm Fit: Results of simple linear regression to predict WCS at post, 6-months follow-up, and at 12-months follow-up based on number of completed session

Variable	df	Parameter estimate (SE)	95% CI	*t* statistic	*p*
**WCS Baseline – Post, n = 294**					
Intercept	1	2.25 (3.62)	-4.86, 9.37	0.62	.53
Completed sessions	1	-0.86 (0.54)	-1.48, -0.23	-2.71	.007
**WCS Baseline – 6-months follow-up, n = 160**					
Intercept	1	-0.11 (6.24)	-12.45, 12.22	-0.02	.99
Completed sessions	1	-0.86 (0.54)	-1.92, 0.20	-1.60	.11
**WCS Baseline – 12-months follow-up, n = 111**					
Intercept	1	12.40 (6.69)	-0.86, 25.66	1.85	.07
Completed sessions	1	-2.04 (0.58)	-3.19, -0.90	-3.54	<.001

Note. Abbreviations: WCS, Weight and Shape Concerns Scale

Adverse events

Table S22

Absolute and relative frequency of adverse events per study arm and assessment time point

	Adverse events, *n* (%)				
**Study arm**	**Baseline**	**Mid-intervention**	**Post**	**6-month follow-up**	**12-month follow-up**
Basic					
AE1	3/391 (0.8)	Not assessed	2/151 (1.3)	4/74 (5.4)	3/57 (5.3)
AE2	1/61 (1.6)	Not assessed	1/27 (3.7)	0/15 (0)	0/11 (0)
AE3	9/452 (2.0)	Not assessed	7/178 (3.9)	5/89 (5.6)	3/68 (4.4)
AE4	0/452 (0)	Not assessed	0/178 (0)	0/89 (0)	0/68 (0)
Original					
AE1	8/259 (3.1)	2/68 (2.9)	1/60 (1.7)	4/29 (13.8)	2/24 (8.3)
AE2	13/137 (9.5)	2/44 (4.5)	1/37 (2.7)	2/12 (16.7)	3/12 (25)
AE3	1/396 (0.3)	0/112 (0)	1/97 (1.0)	1/41 (2.4)	1/36 (2.8)
AE4	0/396 (0)	0/112 (0)	0/97 (0)	0/41 (0)	0/36 (0)
Plus					
AE1[a]	-	-	-	-	-
AE2	244/1387 (17.6)	60/384 (15.6)	43/303 (14.2)	16/146 (11.0)	10/100 (10)
AE3	1/1387 (0.1)	0/384 (0)	1/303 (0.3)	0/146 (0)	1/100 (1)
AE4	2/1387 (0.1)	0/384 (0)	0/303 (0)	0/146 (0)	1/100 (1)
AN					
AE1	1/72 (1.4)	4/25 (16)	1/22 (4.5)	0/13 (0)	1/9 (1.1)
AE2	0/8 (0)	0/2 (0)	0/0 (0)	0/0 (0)	0/0 (0)
AE3	0/80 (0)	0/27 (0)	1/22 (4.6)	2/13 (15.4)	2/9 (22.2)
AE4	0/80 (0)	0/27 (0)	0/22 (0)	0/13 (0)	0/9 (0)
Fit					
AE1	14/1105 (1.3)	17/382 (4.5)	5/242 (2.1)	7/133 (5.3)	2/97 (2.1)
AE2	20/234 (8.5)	4/86 (4.7)	2/53 (3.8)	0/27 (0)	0/16 (0)
AE3	0/1339 (0)	0/468 (0)	0/295 (0)	0/160 (0)	0/113 (0)
AE4	0/1339 (0)	0/468 (0)	0/295 (0)	0/160 (0)	0/113 (0)

Note. [a] AE1 is an inclusion criterion for study arm Plus, therefore no new onsets of symptoms were recorded.

Abbreviations: AE1, adverse event 1, onset of binge eating and/or compensatory behaviors between screening and follow-up-period in participants who did not show these symptoms at screening; AE2, adverse event 2, diagnostic threshold of frequency of binge eating and/or compensatory behaviors is met during the follow-up-period by participants with prior subthreshold symptoms; AE3, adverse event 3, BMI drops below 18.0 kg/m^2 during the follow-up-period; AE4, adverse event 4, participant reports inpatient treatment for ED during the follow-up-period.
